# Enhancing reciprocal partner support to prevent perinatal depression and anxiety: a Delphi consensus study

**DOI:** 10.1186/s12888-016-0721-0

**Published:** 2016-02-03

**Authors:** Pamela Pilkington, Lisa Milne, Kathryn Cairns, Thomas Whelan

**Affiliations:** School of Psychology, Faculty of Health Sciences, Australian Catholic University, Locked Bag 4115, Fitzroy, Victoria 3065 Australia; Melbourne School of Population and Global Health, University of Melbourne, 207 Bouverie Street, Melbourne, Victoria 3010 Australia

**Keywords:** Anxiety, Delphi, Depression, Partner, Perinatal, Prevention, Support

## Abstract

**Background:**

Systematic reviews have established that partner support protects against perinatal mood problems. It is therefore a key target for interventions designed to prevent maternal and paternal depression and anxiety. Nonetheless, the extant literature is yet to be translated into specific actions that parents can implement. Prevention efforts aiming to facilitate reciprocal partner support within the couple dyad need to provide specific guidance on how partners can support one another to reduce their vulnerability to perinatal depression and anxiety.

**Method:**

Two panels of experts in perinatal mental health (21 consumer advocates and 39 professionals) participated in a Delphi consensus study to establish how partners can support one another to reduce their risk of developing depression and anxiety during pregnancy and the postpartum period.

**Results:**

A total of 214 recommendations on how partners can support each other were endorsed by at least 80 % of both panels as important or essential in reducing the risk of perinatal depression and anxiety. The recommendations were grouped under the following categories: becoming a parent, supporting each other through pregnancy and childbirth, communication, conflict, division of labor, practical support, emotional support, emotional closeness, sexual satisfaction, using alcohol and drugs, encouraging self-care, developing acceptance, and help-seeking.

**Conclusion:**

This study established consensus between consumers and professionals in order to produce a set of guidelines on how partners can support each other to prevent depression and anxiety during pregnancy and following childbirth. It is hoped that these guidelines will inform the development of perinatal depression and anxiety prevention efforts.

**Electronic supplementary material:**

The online version of this article (doi:10.1186/s12888-016-0721-0) contains supplementary material, which is available to authorized users.

## Background

### Burden and prevalence

Perinatal distress is a significant public health problem that negatively impacts the individual [1], compromises the partner relationship [2], and can have significant deleterious effects on the child’s development [3]. Depression and anxiety are common among parents during the perinatal period, with meta-analyses indicating that perinatal depression affects 12.9 of mothers [4] and 10.0 % of fathers [5]. Given the reluctance of some new parents to disclose that they are experiencing mood problems, the prevalence of perinatal distress is likely to be underreported [6–8]. The high prevalence of perinatal depression and anxiety and low rates of help-seeking [6] indicate a need for effective prevention approaches that target all parents [9]. The current study aimed to identify how we can enhance partner support and relationship satisfaction for both partners in the couple dyad, to reduce men and women’s vulnerability to perinatal mood problems. We deliberately aimed to be inclusive of mothers and fathers in both opposite-sex and same-sex relationships.

### A couples-based approach to preventing perinatal depression and anxiety

Partner support is a key target for prevention interventions for perinatal depression and anxiety as it is an established modifiable protective factor [10–13]. The transition to parenthood is a significant stressor for both parents [14] that can result in increased marital conflict and decreased marital quality [15]. Preventive interventions that aim to promote parents’ mental health and well-being should help partners support each other in adjusting to this significant life event [16].

A couples-focused approach recognizes that both maternal and paternal depression and anxiety need to be addressed [14]. Research suggests that partners’ mental health is interrelated [17]. Up to 50 % of fathers with a partner experiencing postpartum depression will also develop symptoms, often following the onset of postpartum depression in their partner [18]. Paternal distress can have adverse effects on infants’ emotional and behavioral development [19], particularly if the mother is also depressed [20]. Longitudinal research suggests that the correlation between maternal and paternal postpartum depression is mediated by partner support and relationship satisfaction [14].

Despite these findings, fathers report that the potential for them to develop postnatal depressive symptoms is often not recognized and their capacity to provide support to their partners is minimized by healthcare professionals [21]. Although societal changes in gender roles has seen fathers’ involvement in parenting increase, health services continue to focus on the well-being of mothers [20, 22]. The potential for fathers to develop perinatal mood problems needs to be recognized given the reciprocal nature of the couple relationship and the correlation between partners’ mental health [17]. Prevention efforts should also be inclusive of same-sex parents. Heterosexual and same-sex parents share more similarities than differences in their experience of parenthood, including common risk factors for perinatal mood problems [23].

### Parents have limited knowledge about perinatal depression and anxiety

Most expectant parents are open to learning new information [24] and extensive resources on pregnancy and the experience of childbirth are readily available. In contrast, parents’ understandings of perinatal mental health problems are often more limited [11]. Antenatal classes tend to focus on childbirth and may not adequately prepare couples for the emotional adjustment to parenthood. A number of researchers and policymakers have identified the need for new parents to improve their perinatal mental health literacy e.g., [11]. Improved awareness of perinatal depression and anxiety is likely to lead to earlier recognition of symptoms and increased help-seeking [25, 26].

Existing partner-inclusive interventions aiming to prevent perinatal depression and anxiety mostly involve face-to-face psycho-education delivered by professionals [27, 28]. The reliance on professionals limits the scalability and accessibility of these interventions, and overlooks evidence that parents prefer to access support from family [21, 29]. There is a need for universal prevention efforts that nurture parents’ sense of self-efficacy by providing them with knowledge about the actions *they* personally can take to reduce their partner’s risk [30].

Parents who feel that they have personal control over their risk of perinatal depression are less likely to develop symptoms [31]. Providing expectant parents with information on the specific actions they can take to prevent perinatal mood problems is likely to increase their sense of empowerment. In particular, interventions that promote interpersonal agency - the achievement of positive outcomes through interactions with others [32] - are likely to be beneficial [31]. Prevention efforts should therefore empower parents with specific guidance on how they can support each other to reduce their risk of perinatal depression and anxiety, without necessitating face-to-face professional intervention, which is resource-intensive and difficult to scale [33].

### Parents need specific guidance on how best to support each other

Rowe, Holton [21] found that couples are unsure about the precise ways they can meet each other’s emotional needs following childbirth. Wee et al. [34] argue that the most effective support for fathers is likely to come from their partners, with more than 90 % of men seeing their partners as an important source of emotional or informational support during pregnancy [29]. Letourneau, Duffett-Leger [25] noted that mothers also see partners as their primary source of support, but that their partners’ understanding of how to offer support is limited. A survey of expectant fathers confirmed that a significant proportion of men worry about their capacity to adequately support their partner, “losing” their partner to the baby, and maintaining closeness [29]. Rosenquist [35] argues that although partners are encouraged to monitor and recognize symptoms of depression, this needs to be supplemented with an understanding of how to provide support.

Our systematic review and meta-analysis of factors that are associated with perinatal depression and anxiety that couples can modify [36] identified that there is sound evidence that emotional closeness and partner support protect against both depression and anxiety. There was also strong evidence that positive communication and emotional and instrumental support protect against perinatal depression, while inter-partner conflict is a significant risk factor. Although empirical studies show that aspects of partner support and perinatal depression and anxiety are linked, the literature mostly relies on self-report measures, that are difficult to translate into specific actions that parents can implement [37]. To effectively improve partner support, interventions need to provide couples with specific guidance [37].

### The current study

The current article reports the findings of a Delphi consensus study on the actions that partners can take to reduce each other’s risk of developing depression and anxiety during the transition to parenthood. We included both depression and anxiety as outcomes, as targeting both concurrently is more likely to be effective and less costly [28]. Similarly, we focused on both antenatal and postnatal outcomes, given evidence that antenatal depression is equally as common as postnatal depression [38, 39], and anxiety peaks in the last trimester of pregnancy [40]. Moreover, co-morbid depression and anxiety may be more treatment resistant [41], and is more strongly associated with lack of parental warmth than anxiety alone [42]. The resulting recommendations can be promoted to new and expectant parents to help prevent perinatal depression and anxiety.

## Methods

### The Delphi method

The Delphi method [43] was used to establish expert consensus on the actions that partners can take to prevent each other from developing perinatal depression and anxiety. The Delphi technique was first developed in the 1950s by the United States government to inform military decisions [44], but is now widely-used in health research to inform policy and service planning, develop clinical guidelines, and identify professional competencies [45, 46]. Delphi studies are increasingly implemented via the internet, and a number of researchers have drawn on the experience and expertise of consumers and other key stakeholders to develop mental health promotion guidelines using this method e.g., [47–49]. See [46] for an overview of the use of the Delphi expert consensus method in mental health research.

A panel of experts independently rated the extent to which they believed each action to be important for the prevention of perinatal depression and anxiety. The ratings were made over three successive rounds. Following each round, panel members were provided with summaries of the findings from the previous round, and asked to consider whether they would like to change or maintain their original rating. The study was reviewed by the Human Research Ethics Committee at the Australian Catholic University (No. 2013_246V).

### Panel formation

Two expert panels were formed of [1] researchers and clinicians with a minimum of five years of experience in perinatal mental health (professionals), and [2] consumer and carer advocates with lived experience of perinatal depression or anxiety (consumers). A minimum of five years was required to ensure that professionals had sufficient expertise and experience. Consumer advocates were required to (a) have suffered from perinatal depression or anxiety or cared for someone who had; (b) be currently well; and (c) be in a consumer advocacy role (e.g., peer support, public awareness). All participants were required to be aged 18 years or older.

Panel members were recruited internationally by emailing an advertisement to relevant individuals, professional groups, and organizations. The following sampling pools were used to identify potential participants: (1) authors of articles in our systematic review and meta-analysis of factors associated with perinatal depression and anxiety that couples can modify [36]; (2) psychologists listed on the Australian Psychological Society’s *Find a Psychologist* website (http://www.psychology.org.au/FindaPsychologist) who identified their area of expertise as “Depression/Anxiety” and “Couples”; (3) consumer and carer advocacy organizations and websites (e.g., the Post and Ante Natal Depression Association); (4) professional organizations and websites (e.g., the International Marcé Society for Perinatal Mental Health); and (5) individuals known to the authors to have relevant clinical or research experience.

### Questionnaire development

We systematically searched websites and books to identify actions that parents could take to prevent depression or anxiety in their partner. The search for websites was conducted on 1 August 2014 by entering the search terms “prevent* (depression OR anxiety) (partner OR couple OR relationship) (postpartum OR postnatal OR antenatal)” into five search engines (http://google.com, http://google.ca, http://google.com.au, http://google.co.nz, http://google.co.uk). The top 50 websites identified by each search engine were captured using *NCapture* [50], a web browser extension for Google Chrome that captures web pages for analysis in *NVivo 10* [51]. Duplicate webpages were deleted. If sites referred to other relevant webpages or books, these were also obtained and screened. Further books were identified by entering the search terms “prevent depression pregnancy postpartum” into Amazon (http://www.amazon.com). Inclusion of “anxiety” as a search term did not produce additional results. The search identified 15 books and 358 webpages. The academic literature was also searched for relevant partner support strategies via three electronic databases (PsycInfo, MEDLINE and CINAHL) using the search terms “(partner or spouse or couple) and (depressi* or distress or affective or mood or anxiety or PND) and (postpartum or postnatal or perinatal or antenatal or pregnancy)”. The academic literature did not contribute any items but formed the basis of the evidence summaries provided to panel members (see below).

The first author screened the search results for suggestions on how couples can support one another to prevent perinatal depression or anxiety using *NVivo 10* [51]. *NVivo 10* enables the user to import web pages as pdfs and code the text into categories. The nine themes that emerged from our systematic review and meta-analysis [36] (supporting each other through pregnancy and childbirth, communication, conflict, division of labor, practical support, emotional support, emotional closeness, sexual satisfaction, using alcohol and drugs) were used as a priori categories. In addition, four new categories emerged from the lay literature (becoming a parent, encouraging self-care, developing acceptance, help-seeking). These categories were formulated by the first author, and refined through discussion with the co-authors. This process identified 1253 suggestions that were drafted into individual questionnaire items by the first author. Items that were repetitive in content were consolidated into single items that captured the central idea. Suggestions involving more than one idea were divided into multiple items. Most sources framed suggestions in terms of how fathers can support mothers. When possible, suggestions were reworded to be gender-neutral (e.g., “Partners should do things to show each other love and appreciation, e.g., buy flowers, make a cup of tea, give massages”) so that they applied to both heterosexual and same-sex couples, unless the suggestion was explicitly gender-specific (e.g., “Partners should help the child-bearing mother with heavy lifting and carrying as much as possible”). The authors formed a working group to screen the items to ensure that they were actionable by partners, comprehensible, and represented all the ideas identified by the literature search. The final questionnaire comprised 252 items from 210 webpages and four books.

### Questionnaire administration

Panel members rated the importance of each item for preventing the development of perinatal depression or anxiety. The rating scale was 1 = *Essential*, 2 = *Important*, 3 = *Don’t know/Depends*, 4 = *Unimportant*, 5 = *Should not be included*. Panel members were instructed to base their ratings on any knowledge available to them, including research evidence, clinical experience in treating individuals with perinatal mental health problems, lived experience of perinatal depression or anxiety, or experience with caring for someone with perinatal depression or anxiety. Where available, brief summaries of the academic literature reviewed in Pilkington, Milne [36] were included in the questionnaire for panel members to consider when deciding their ratings.

The questionnaire also included definitions for the following key terms. *Perinatal depression* was defined as a non-psychotic major depressive episode occurring during pregnancy or the first 12 months after childbirth [52]. *Perinatal anxiety* was defined as the presence of anxiety symptoms occurring during pregnancy or the first 12 months after childbirth. *Partner* was defined as an adult, with whom the mother or father of the infant shares an intimate relationship, including de facto and same-sex relationships. The term *primary caregiver* refers to the individual who takes primary responsibility for caring for the baby.

The questionnaire was administered on-line over three rounds using *Qualtrics* survey software [53]. Respondents were sent up to three email reminders per round. Those who completed less than 50 % of a survey round were excluded from the subsequent round/s. The Round 1 questionnaire consisted of items identified from the literature search. The questionnaire responses were analyzed to determine which items were endorsed by panel members as important for preventing perinatal depression and anxiety. Items that did not establish clear consensus in Round 1 were re-rated in Round 2*.* Panel members were also invited to suggest additional statements not included in the Round 1 questionnaire, to be rated in Round 2. Suggestions judged to be a new idea were drafted into items and included in the Round 2 questionnaire.

The Round 2 questionnaire therefore consisted of 1) new items to be rated for the first time, and 2) items that did not achieve clear consensus in Round 1 and needed to be re-rated. Items from Round 1 that were re-rated in Round 2 and did not achieve sufficient consensus a second time were excluded. New items in Round 2 that did not establish clear consensus were re-rated in the third and final round. Members of the panel were not given the opportunity to suggest additional statements in Round 2. Therefore, Round 3 consisted solely of new items from Round 2 that required re-rating.

### Statistical analysis

The questionnaire responses were analyzed to determine the percentage of panel members who endorsed each item as important for preventing perinatal depression and anxiety. Following the conventions of similar Delphi consensus studies e.g., [54] the following cut-off points were used:

- Included items: The item was included in the final guidelines if it was endorsed by at least 80 % of both panels (professionals and consumers) as either Important or Essential.
- Re-rated items: The item was re-rated in the subsequent round if it was endorsed by at least 80 % or more of one of the two panels as Important or Essential OR it was endorsed by 70 to 79 % of both panels as Important or Essential. Items were re-rated once only.
- Excluded items: Items that did not meet the above criteria were excluded.

Following each survey round, panel members were provided with a summary report that listed which items had been endorsed and excluded, and which items needed re-rating. For those items that needed to be re-rated, the report listed the percentage of consumers and professionals that had endorsed the item, and the participant’s personal rating. This was to allow participants to compare their ratings with the other panel members’ and choose whether they wished to maintain or change their rating in the subsequent round.

## Results

### Panel members

The invitation email was sent to 1191 individuals and 50 organizations. Thirty-six emails returned non-delivery reports and 101 returned “out of the office” replies. Of the 1054 individual emails that were successfully sent, 136 individuals (13 %) started the survey. Of these, 60 experts (39 professionals, 21 consumers) completed at least 50 % of the Round 1 questionnaire, yielding a response rate of 44 %. The majority of panel members (88) responded to all Round 1items. There was some attrition over the course of the three questionnaire rounds. The Round 2 survey was completed by 13 consumers (62 of Round 1), and 26 professionals (67 %). Twelve consumers (92 of Round 2) and 21 professionals (81 %) completed the final Round 3 survey. These unequal panel sizes do not influence the results, as equal weight is given to the ratings of each panel.

### Professionals

The professional panel comprised 31 clinicians and eight researchers. The clinicians primarily worked as psychologists (*n* = 15), psychiatrists (*n* = 5), and nurses (*n* =3). Social workers, counsellors, and other specialists (*n* = 8) also participated*.* The professionals reported working in a range of contexts, including hospitals, universities, family therapy, private practice, and specialist perinatal mental health services. Most (69 %) had at least 16 years of specialist experience in perinatal mental health. Twenty-four were from Australia, six from the United States of America, four from Europe, two from Canada, two from the United Kingdom, and one from the Middle East.

### Consumer advocates

Consumer advocates were affiliated with organizations such as Post and Ante Natal Depression Support and Information Inc (PANDSI) and Pre and Postnatal Depression Advice and Support (PANDAS). Seventeen were from Australia, two from the United States of America, one from Canada, and one from the United Kingdom.

### Endorsed statements

**Fig. 1:**
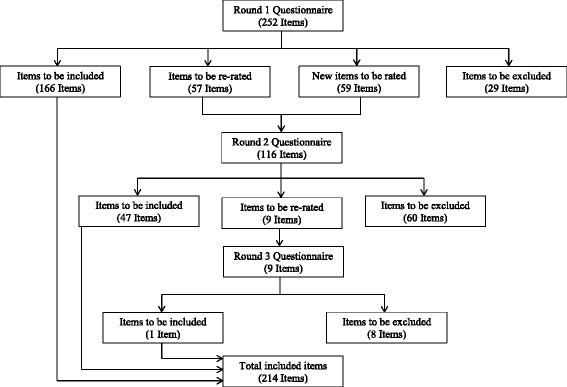
The number of items included, re-rated, and excluded at each round of the questionnaire
- Round 2
  Of the 116 items in Round 2, 47 achieved adequate consensus to be included in the final guidelines, 60 items were excluded, and 9 met criteria to be re-rated in the third and final round.
- Round 3
  In Round 3, one of the nine items achieved adequate consensus to be included in the final guidelines. Items not endorsed in Round 3 were excluded. This produced a total of 214 items for inclusion in the final guidelines as suggestions for how partners can support each other to reduce each other’s risk of developing perinatal depression or anxiety. See On-line Additional file 1 for a full list of the items meeting criteria for inclusion, exclusion, and re-rating at each round.

- Round 1
  Figure 1 shows the number of items that were included, excluded, and re-rated at each round of the survey. In the Round 1 survey, 166 items were rated as essential or important by 80 % or more of both panels, 29 items were excluded, and 57 items met criteria to be re-rated in Round 2. Based on the suggestions made by panel members in Round 1, 59 new items were developed. Of these, 15 were modified versions of items from Round 1.

### Final recommendations

The 214 recommendations were synthesized into a cohesive document by the first author. This involved removing contextual strings from items (“Partners should…”) and adding conjunctions. The content or meaning of the items was not altered. The document was organized based on the survey subheadings. Some of the shorter sections were collapsed together, and the wording of some subheadings was updated (e.g., sexual satisfaction was updated to “Sex and intimacy”), to increase readability. Refer to Table 1 for examples of the endorsed recommendations in each of the questionnaire categories, and how these map onto the subheadings used in the final document.

**Table 1:** Examples of partner strategies under the subheadings used in the Delphi questionnaires and the final guidelines

Final document subheadings	Questionnaire categories	Example items
Becoming parents	• Becoming a parent • Developing acceptance	• Identify potential sources of stress, such as relationship problems or financial difficulties, and explore ways of dealing with these problems before the baby is born. • Be willing to continually explore and adapt, as what works one day may not work the next
Pregnancy and childbirth	• Supporting each other though pregnancy and childbirth^a^	• Share how you are feeling about labour and childbirth during pregnancy
Tips for communicating	• Communication^a^	• Share your concerns, thoughts, and feelings with each other
Managing conflict	• Conflict^a^	• Use ‘I’ statements, e.g., Instead of saying, “You don’t make any time for us anymore”, say “I feel lonely when we spend less time together”
Sharing the workload	• Division of labor^a^	• Plan the division of labour and agree on who does what before the baby is born, e.g., talk about who will be employed in paid work
Seeking help from family and friends	• Practical support^a^	• Discuss and consider what supports you will draw on when you become parents
Showing affection and acceptance	• Emotional support^a^	• Validate each other’s thoughts, experiences, and worries, e.g., “I can see how hard this is for you”, “This would be a hard time for anyone”, “You have been dealing with so much lately”
	• Emotional closeness^a^	• Do what you can to strengthen your connection during pregnancy and following childbirth, e.g., let each other know that you love each other
Sex and intimacy	• Sexual satisfaction^a^	• If you or your partner lose interest in sex, explore different types of intimacy, such as cuddling or hand holding
Staying healthy	• Encouraging self-care • Using alcohol and drugs^a^	• Look for quick and easy meal options that incorporate lean meats, whole-grains, low-fat dairy products and fresh fruit, and vegetables • Be aware that there are healthier ways of coping than alcohol or drug use
Seeking help	• Help-seeking	• Encourage your partner to seek professional help if you think she or he is experiencing depression as this will benefit their health, the healthy development of your baby, and your relationship

^a^Accompanied by summary of evidence

The working group edited the document to ensure that it was coherent and that it maintained fidelity to the items endorsed by the expert panels. Panel members were then invited to provide feedback on the wording and structure of the draft guidelines. Panel member comments judged by the working group as improving the comprehensibility of the guidelines, without introducing a new idea, were integrated into the final document. The document was then formatted by a graphic designer for dissemination to new and expectant parents. The final guidelines are provided in On-line Additional file 2 to be freely reproduced for non-profit purposes, provided the source is acknowledged.

### Differences between consumers and professionals

Post-hoc analyses were conducted to explore differences in the extent to which each panel endorsed the items. Overall there was considerable agreement about the strategies considered important to the prevention of perinatal depression and anxiety (*r* = .73, *p* < .05). In Round 1, the professional and consumer panels’ responses provided similar levels of endorsement in between 72 to 80 % of instances (i.e., whether the item should be included, excluded, or re-rated). Items that differed in the level of endorsement by more than 20 % are provided in Table 2.

**Table 2:** Partner strategies^a^ with large differences in endorsement between panels

Strategy	Endorsed by consumers as “Essential” or “Important” (%)	Endorsed by professionals as “Essential” or “Important” (%)	Difference (%)
• If their partner is experiencing problems with anxiety, partners should encourage them to consider taking supplements such as magnesium and calcium, as these are effective in reducing anxiety	71.4	33.3	38.1
• If their partner is resistant to going out, partners should think of things that they can do together in the home that give them a break from parenting, e.g., board games, watching a movie	95.2	67.6	27.6
• Partners should be aware that there is very little they can do to help the child-bearing mother during labor	47.6	20.5	27.1
• Partners should help with the cleaning	90.5	63.9	26.6
• Partners should help with housework before having to be asked by the primary caregiver	95.2	69.4	25.8
• Partners should try to get outdoors together with the baby as much as possible	90.5	64.7	25.8
• Partners should help the primary caregiver with preparing meals, e.g., food shopping, cooking, clearing the table	90.5	66.7	23.8
• Partners who are working should telephone their partner from work, or drop in for lunch occasionally if they work close to home	76.2	52.9	23.2
• Partners should monitor each other for withdrawal or change in mood	85.7	62.5	23.2
• If their partner is experiencing depression, partners should also seek professional help for themselves	85.7	62.5	23.2
• Partners should be prepared to listen even if they feel that they are hearing the same things over and over	100.0	76.9	23.1
• Partners should challenge negative thinking by pointing out situations or tasks that their partner has handled well	95.2	74.3	21.0
• Partners should set aside quiet time to spend together while the baby is sleeping, even if it is only for 10 min	100.0	79.4	20.6

^a^Strategies with at least a 20 % difference in endorsement

## Discussion

The aim of this study was to identify how partners can support each other over the perinatal period in order to reduce their risk of developing depression or anxiety. The research literature has identified partner support as a key protective factor against mood problems during pregnancy and following childbirth. Using the Delphi consensus method this study has translated this evidence base into specific, actionable recommendations on *how* partners can reduce each other’s risk of developing depression and anxiety.

We identified 214 recommendations grouped under 13 categories, nine of which corresponded to partner factors in our systematic review and meta-analysis of risk and protective factors associated with perinatal depression and anxiety that partners can potentially modify. Partner factors that were supported with sound evidence in our systematic review (i.e., positive communication, emotional closeness, emotional support, practical support and minimizing conflict) were also widely endorsed by panel members. However, a number of items relating to practical support received only qualified endorsement. Items relating to accessing practical support from sources outside of the family (i.e., friends, parent groups, on-line forums, support groups, play groups and workmates) were endorsed by very few respondents. This is consistent with qualitative research suggesting that partners prefer to access support from each other and other family members [21] and reinforces the need for prevention interventions that enhance partners’ ability to support one another. It may also reflect panel member’s perceptions that couples’ sources of support vary greatly depending on their individual circumstances. This is confirmed by the finding that 100 % of both the professional and consumer panels endorsed the item “Partners should discuss and consider what supports they will draw on when they become parents”.

Of note, a number of categories without supporting literature nonetheless also received widespread endorsement. In particular, all of the items on developing acceptance (e.g., “Partners should try to enjoy their family rather than feel that they are missing out on the old days”) were highly endorsed by both panels. Although our systematic review of partner factors associated with perinatal depression and anxiety did not identify literature relating to this category, the results are consistent with evidence that incongruence between expectations and the reality of parenthood is common in men [55] and women [52] experiencing postpartum depression. An accepting and flexible attitude towards the pressures of early parenthood may therefore be protective against depression and anxiety. Items relating to how partners can encourage each other to take care of themselves (e.g., by being physically active) were also widely endorsed. Finally, suggestions relating to satisfaction with the sexual relationship were largely supported, even though the evidence base for this is only emerging [36]. It appears that these areas are under-researched and warrant further investigation.

Generally, there were high levels of agreement between the consumer advocate and professional panels. Items that were endorsed by 100 % of both panels mostly related to the need for relationship and mental health problems to be “taken seriously” and professional help sought when needed (e.g., “Partners should take their partner seriously if she or he talks about not wanting to live or about harming themselves”). Items relating to awareness that the perinatal period is stressful and it is normal to experience a wide range of emotions were also endorsed by 100 % of both panels. It is clearly seen as important for partners to have the capacity to differentiate between the normal stress that is part of becoming a parent and problems that require professional intervention.

There were some large differences between the two panels in their endorsement, particularly of the recommendations on practical support. Consumers tended to highly endorse these items, while professionals were less likely to indicate that they should be included in the final guidelines. For example, 90 % of the consumer panel endorsed the item that “Partners should help with the cleaning” while only 64 % of the professional panel believed this was important. This is surprising given the sound research evidence that practical support protects against perinatal depression [36]. This finding could reflect that professionals are more likely to work with individuals experiencing more severe symptoms of perinatal depression and anxiety, and therefore the relevance of practical support is not as salient. In contrast, individuals with personal experience of perinatal depression would be more likely to endorse the need for practical support [25].

Another surprising finding was that none of the items relating to both mothers and fathers attending antenatal appointments and classes were endorsed. Attendance by both partners does not appear to be seen as relevant to the prevention of perinatal depression and anxiety. Alternatively, panel members’ reluctance to endorse antenatal classes may reflect that perinatal health services are currently focused on maternal well-being. Antenatal classes are usually only attended by one of the partners [56], most commonly the child-bearing mother [57], as fathers often have limited availability due to work commitments. Alternative formats such as web-based interventions, as well as men’s preferences around the format and timing of interventions, need to be considered to optimize accessibility for both men and women [27].

### Strengths and limitations

The study utilized a well-established method that is widely used to develop mental health promotion guidelines. The inclusion of consumer and carer advocates as experts is a key strength of this study. Consumer involvement improves the relevance and usefulness of mental health promotion guidelines [58]. There was attrition between rounds but this occurred at a similar rate to other Delphi consensus studies [47, 48, 59]. Future research should consider potential strategies to improve responses rates, such as the use of incentives. The time commitment involved in Delphi studies is also likely to contribute to attrition rates, and efforts should be made to minimize the participant burden. Even so, the number of participants is less relevant to the validity of Delphi studies than the appropriateness and credibility of the panel members [60]. The panel members had considerable and diverse expertise, and more than two-thirds of the professional panel had over 16 years’ experience in perinatal mental health. The use of successive rounds further increases the content validity of the Delphi findings.

Although relatively few consumer advocates participated in the study, and all but one were female, any potential bias from the difference in panel size was overcome by giving equal weight to the ratings of each group. As we did not ask members of the consumer advocate panel to specify whether they were in heterosexual or same-sex relationships, further research is needed to confirm that same-sex couples see the recommendations as relevant.

The results of this study may not apply to all couples, depending on their cultural background and individual circumstances. The relevance of the guidelines is limited to parents who are in a relationship with a partner. The findings may not generalize to other family constellations, such as blended families, or families in which the grandparent has a primary role in the care of the grandchildren. Further research is needed to establish the relevance of the guidelines to diverse family types, such as couples who have adopted their children, who may face unique stressors. Given that social support is well-established as a protective factor against perinatal distress [61–64], it could also be useful to expand the current research to consider how other people in the parent’s social network, such as their family of origin, can be supportive. Nonetheless, at least 84 % of families in Australia are two-parent families [65], and panel members were reluctant to endorse items around seeking support from friends and family.

Although we recruited an international panel, a number of respondents raised concerns that the study was prescriptive and did not sufficiently acknowledge the diversity of families or take individual circumstances into account. As noted by Reavley, Ross [54], one of the challenges of developing guidelines is to make them specific enough to be useful while remaining broad enough to be relevant to most people. The guidelines are designed to be generalizable to most couples as it is hoped that the final guidelines will inform the development of universal prevention efforts. Some panel members also questioned whether certain items pathologized parenthood, an issue that has received attention in the academic literature e.g., [66]. We attempted to counter this by phrasing items positively (i.e., what partners could do, rather than what they should not do).

### Future directions

Further research is needed to evaluate whether the provision of these guidelines as an information booklet translates into enhanced support, improved relationship satisfaction, and reduced vulnerability to perinatal depression and anxiety. A more dynamic and engaging format, such as a website, may be more effective in eliciting behavior change [67]. Future research should translate the recommendations into an intervention format that is appealing to new parents, and evaluate their efficacy in preventing perinatal depression and anxiety. Consultation with new parents, such as through focus group discussions, could elucidate preferred formats for dissemination.

Consideration of the range of complex factors that influence whether support is effective, or has unanticipated negative consequences (e.g., the recipient feeling incompetent [37]), was outside the scope of the current research. However, future research should also explore these and other factors (e.g., work-family conflict) that influence partners’ capacity to support one another [68]. Finally, given that partner support is a protective factor against various adverse outcomes, in addition to mood problems, future research could investigate whether enhancing partner support influences other aspects of perinatal well-being such as co-parenting and parent–child relationships [69].

## Conclusions

This study has identified a set of freely available recommendations (Online Additional file 2) that are supported by the expert opinion of perinatal mental health clinicians and researchers, as well as consumer and carer advocates with lived experience of perinatal depression and anxiety. These guidelines can be promoted to new and expectant parents to help them understand how they can best support each other to protect themselves from depression and anxiety. It is also hoped that these guidelines will inform the development of prevention interventions for perinatal depression and anxiety that target the couple relationship, either as the focus of the intervention or alongside other risk and protective factors.

## Additional files

Additional file 1: Items meeting criteria for inclusion, exclusion, and re-rating at each round.(DOCX 41 kb) Additional file 2: Final guidelines on how partners can support one another to prevent perinatal depression and anxiety. (PDF 1173 kb)
