# Supplementary material for: Enhancing reciprocal partner support to prevent perinatal depression and anxiety: a Delphi consensus study
Source: BMC Psychiatry. 2016 Feb 3;16:23. doi: 10.1186/s12888-016-0721-0 (PMC4739319; doi:10.1186/s12888-016-0721-0)
Supplement: Additional file 1: — Items meeting criteria for inclusion, exclusion, and re-rating at each round.(DOCX 41 kb) [file 12888_2016_721_MOESM1_ESM.docx]

**Online Supplement 1**

*Items meeting criteria for inclusion, exclusion, and re-rating at each round*

| Round | Included (Endorsed by >80% of both panels) | To be re-rated (Endorsed by >80 of one panel or >70 <79% of both panels) | Excluded |
| --- | --- | --- | --- |
| Round 1 | 1. Partners should be aware that it is normal to experience a wide range of emotions during pregnancy and the following year 2. Partners should be aware that even when things are progressing normally, pregnancy and new parenthood can put stresses on them, their partner and their immediate family 3. Partners should be aware that most couples ﬁnd the transition to parenthood challenging 4. Partners should be aware that services are available to help with the challenging aspects of parenting, e.g., Maternal Child Health Nurses, Early Parenting Centres, Baby Sleep Clinics 5. Partners should be aware that their daily routines will change 6. Partners should be aware that having a baby causes changes which may involve a degree of loss and grief, e.g., loss of freedom, work identity, ﬁnancial independence and social contacts 7. Partners should be aware that their partner acting out of character may indicate that they need help adjusting to parenthood 8. Partners should be aware that caring for a baby may place additional stress on their relationship 9. Partners should be aware that when a baby arrives, the focus shifts from self and partner-care to mostly baby-care 10. Partners should learn about the changes in roles, responsibilities, and relationships that occur after birth 11. Partners should think about the sort of partner and parent they want to be and work out how they can achieve this 12. Partners should discuss their parenting hopes, fears, and roles prior to the birth of a baby 13. Partners should communicate their expectations about parenting and try to ensure that their expectations are compatible and realistic 14. Partners should be aware of unrealistic expectations that can cause rifts in their relationship, e.g., I’ll have the same time available for work or leisure activities as before the baby was born 15. Partners should be aware that they bring separate past experiences, ideas and hopes to the new family, and combining these can be difficult 16. Partners should consider their own childhoods and discuss how this might influence what they do and say as parents, e.g., if their partner was not parented warmly themselves, they may need encouragement to spend time with their baby 17. Partners should try to identify what’s important to them - e.g., communicating with others, affection, fun, safety, financial security, time together as a family and so on - and discuss how they can meet these needs 18. Partners should identify potential sources of stress, such as relationship problems or ﬁnancial difﬁculties, and explore ways of dealing with these problems before the baby is born 19. Partners should be aware that symptoms of depression and anxiety are common, particularly among new parents 20. Partners should know about the warning signs of depression and anxiety so that they can seek help early 21. Partners should know the differences between depression and the baby blues 22. Partners should know the risk factors and symptoms of depression 23. Partners should know the risk factors and symptoms of anxiety 24. Partners should be aware that perinatal depression and anxiety can affect both men and women 25. Partners should be aware of the symptoms of depression that may be more typical of fathers 26. Partners should be aware that fathers can get depressed even if their partner is not depressed 27. Partners should be aware that pregnancy is a powerful and life-changing experience for mothers and fathers 28. Partners should encourage the child-bearing mother to rest, particularly if she is having a difficult pregnancy 29. Partners should be aware that childbirth can provoke anxiety 30. Partners should be aware that mothers' and fathers' experiences around pregnancy, labour, childbirth, and early parenthood can be very different 31. Partners should be aware that, unlike the child-bearing mother, partners do not go through all the physical changes of pregnancy and giving birth, so they may not begin to adjust to parenthood until the baby is born 32. Partners should not assume that because childbirth is a natural process, the child-bearing mother will be able to cope without support 33. Partners should share how they are feeling about labour and childbirth during pregnancy 34. Partners should discuss their birth plan and intentions for labour in advance, e.g., talk about whether or not the child-bearing mother wants the partner to be present during the labour 35. Partners should be aware that some degree of stress and fatigue is normal following childbirth 36. Partners should be aware that if the birth was traumatic, it may impact on their sexual relationship 37. Partners should be aware that if the birth was traumatic it may increase their risk of developing depression and anxiety 38. Partners should be aware that babies develop well if their parents can relate to each other with respect and affection 39. Partners should be aware that open communication will strengthen their relationship 40. Partners should be aware that when their partner wants to talk, they’re not necessarily seeking advice, but just need to talk it through 41. Partners should listen to each other’s concerns 42. Partners should ask each other to explain or give more information if they don’t understand what’s being said 43. Partners should actively listen to help each other feel / acknowledged and supported by:-Stopping what they’re doing to show that they are giving their full attention 44. Partners should actively listen to help each other feel / acknowledged and supported by:-Using body language to show that they are listening, e.g., maintaining eye contact and sitting in a relaxed position 45. Partners should actively listen to help each other feel / acknowledged and supported by:-Waiting until the other has finished speaking before offering their opinion or suggestions 46. Partners should be aware that talking is a major part of resolving problems or conflict 47. Partners should share their concerns, thoughts, and feelings with each other 48. Partners should ask each other about their day and how they are feeling, not just focus on the baby, e.g., “What was good?” “What wasn’t as good?” 49. Partners should be mindful of what they say, and how they say it, as it may influence how their partner will respond 50. Partners should voice their needs directly rather than thinking that their partner can read their mind 51. Partners should be aware that their partner may not want to "burden" them with their feelings 52. Partners should not ‘bottle-up’ their feelings as this makes it more likely they’ll come out the wrong way, such as during an argument 53. Partners should be aware that it can be difficult for their partner to find the words to talk about their painful and negative thoughts 54. Partners should encourage each other to talk honestly about what they are struggling with 55. Partners should be careful not to dismiss each other’s concerns when providing reassurance, e.g., rather than saying something isn’t a problem say “I will be with you to help with that” or “I can see that is really worrying you but I think we can get through that" 56. Partners should be aware that professional assistance is available if they need help with communicating effectively, e.g., a couples therapist or a psychologist 57. Partners should be aware that it is common for couples to experience an increase in arguments and tension during pregnancy and following childbirth 58. Partners should manage conflict well as this will benefit their child/children 59. Partners should try to resolve small conflicts before they escalate into major rifts 60. Partners should learn and become aware of their own and their partner’s warning signs that they’re becoming overwhelmed, e.g., clenched jaw, raised voice, door slamming, irritability, indecisiveness 61. Partners should avoid judging each other, e.g., thinking in terms of who is right and who is wrong, or thinking of their partner as the enemy or “the one with the problem” 62. Partners should try to understand their partner’s point of view even if they don’t agree, e.g., “I can understand why you’re angry that I asked my parents over for the weekend without talking to you first" 63. Partners should avoid words or phrases that imply that their partner is always wrong or not trying, e.g., “You always ...” or “You never ..." 64. Partners should use ‘I’ statements, e.g., Instead of saying, “You don’t make any time for us anymore”, say “I feel lonely when we spend less time together" 65. Partners should avoid letting fights continue overnight 66. Partners should take a break if their tempers are too hot and return to communicating when they are calmer, e.g., partners should say something like, “I want to listen to you. I know this is important, but I’m having a hard time because we’re so mad at each other. Can we take a break and talk about it later?" 67. Partners should seek professional help if they are having difficulty resolving their relationship problems 68. Partners should express their needs without criticising each other 69. Partners should avoid name-calling, e.g., "You’re stupid!" 70. Partners should avoid criticising the mother’s body or demanding that she loses weight 71. Partners should avoid making unfavourable comparisons to other parents 72. Partners should give feedback about how they feel when they are criticised by their partner, e.g., “When you do / say ... I feel...” 73. If one partner doesn’t want to talk about the issue at the time it is first raised, partners should schedule another time to discuss it 74. When raising an issue, partners should stay focused on the topic, rather than side track the conversation by raising other issues or concerns 75. Partners should describe what’s causing their concerns without saying why they think it is happening, e.g., instead of saying “You just sit down and watch TV while I have to get the dinner ready and look after the children”, say “I’d find it easier to get dinner if the kids were kept busy. Would you be able to spend some time with them?” 76. Partners should offer suggestions or examples rather than dictating to their partner what to do 77. Partners should review their progress when problem solving by discussing what worked well, what didn’t work, and what they should change 78. Partners should be aware that they can’t always pre-empt or ﬁx everything that goes wrong 79. When problem solving, partners should:-Avoid jumping to conclusions 80. When problem solving, partners should:-Take turns talking 81. When problem solving, partners should:-Try to hear the positive in what their partner is saying 82. When problem solving, partners should:-Avoid trying to force the other person to change 83. Partners should plan the division of labour and agree on who does what before the baby is born, e.g., talk about who will be employed in paid work 84. Partners should be willing to re-negotiate the division of labour as needed 85. Partners should share household chores 86. The primary caregiver should encourage their partner to be involved with the baby and give them space to do this without watching over them, as this will build their confidence and help them build a strong relationship with the child 87. Partners should accept that they may do things differently from each other, and that these different experiences can be good for the baby 88. The partner who is not the primary caregiver should be realistic about what time they'll be home, and come home on time whenever possible 89. Partners who are working should be aware that their partner may feel trapped at home all day and may feel resentful 90. Partners who are feeling nervous or unsure about providing practical support should discuss it with their partner 91. Partners should provide practical support so that the primary caregiver can focus on resting and feeding the baby for the first 6 weeks postpartum 92. Partners who are not the primary caregiver should identify a task that they can make a part of their routine, e.g., bathing the baby 93. If the partner isn’t sure what practical help the primary caregiver wants, they should ask for guidance or make suggestions 94. The primary caregiver should communicate if they need help by specifically stating what they need, e.g., Instead of saying, “I feel overwhelmed and need help around here” ask, “Would you please do the laundry for me this week? I’m feeling so overwhelmed." 95. Partners should try to help out rather than get angry if the primary caregiver is finding it hard to cope with everyday chores 96. The primary caregiver should acknowledge their partner’s practical support 97. Partners should provide the primary caregiver with breaks that they can count on, e.g., taking the baby out for a walk 98. Partners should arrange things so that the primary caregiver has some leisure time at least once a week 99. Partners should take the baby if their partner is getting upset or flustered 100. If they have older children, partners should organise to take turns in caring for the baby so the other can spend one-on-one time with older siblings 101. Partners should help the primary caregiver have time away from the baby doing something they find pleasurable (e.g., a massage or a warm bath) 102. Partners should be aware that existing supports and friendships can change when they become parents 103. During pregnancy, partners should identify support people who will help them following childbirth 104. Partners should be aware that if their partner works away from home, a good support network is particularly important 105. Partners should be aware of pressure and the expectations of others (e.g., parents, in-laws, family, colleagues) and trust their own knowledge and understanding of their baby 106. Partners should discuss and negotiate whether extended family are being supportive or intrusive 107. Partners should tactfully limit visitors and establish boundaries if their partner is becoming overwhelmed by making sure that visitors don’t outstay their welcome or turn up at inconvenient hours 108. Partners should be aware that nurturing each other enables them to nurture the baby 109. Partners should let each other know that they’re there for each other 110. Partners should be patient and understanding with each other 111. Partners should praise each other’s parenting efforts by giving specific examples, e.g., “I love how you smile at the baby” 112. Partners should let each other know if they need more acknowledgement, appreciation and encouragement 113. Partners should validate each other’s thoughts, experiences, and worries, e.g., “I can see how hard this is for you”, “This would be a hard time for anyone”, “You have been dealing with so much lately” 114. Partners should be aware that their partner’s self-esteem may be more fragile after the baby is born 115. Partners should let each other know that they are not failing their baby or partner if they feel stressed 116. Partners should do what they can to strengthen their connection during pregnancy and following childbirth, e.g., let each other know that they love each other 117. Partners should do things to show their partner love and appreciation, e.g., buy flowers, make a cup of tea, give massages 118. Partners should be aware that following childbirth they will have to make a commitment to enjoy some quality alone time, whereas before it was a luxury that they may have taken for granted 119. Partners should arrange to do enjoyable activities together 120. Partners should think about the things they used to like doing before they had the baby and consider how they might do those activities together again 121. Partners should be aware that their sex life is likely to change during pregnancy and following childbirth and may not return to normal for a year or more 122. Partners should be aware that many mothers will have less interest in sex in the later stages of pregnancy and the months following birth due to hormonal changes and the way they feel about their body 123. Partners should be aware that pregnancy, birth and parenting can affect sexual health and intimacy due to the physical recovery after childbirth, lifestyle changes after the birth, and changes in body image 124. Partners should be aware that their partner may feel rejected or unwanted if they are not interested in sex 125. Partners should be aware that less interest in sex does not mean that their partner is no longer interested in them or attracted to them 126. Partners should communicate what they want and how they feel about sex 127. If partners lose interest in sex, they should explore different types of intimacy, such as cuddling or hand holding 128. Partners should be aware that if their partner is feeling low and has also lost interest in sex, this can be a sign of depression 129. Partners should encourage each other to sleep when needed 130. Partners should look for quick and easy meal options that incorporate lean meats, whole-grains, low-fat dairy products and fresh fruit, and vegetables 131. Partners should choose healthy snacks (e.g., fruit, low-fat yoghurt, raw nuts and seeds, wholegrain crackers) over highly processed foods such as biscuits, cake, and chocolate 132. Partners should encourage each other to be physically active either individually or together 133. Partners should help each other to get out and get some fresh air 134. Partners should be aware that mothers with anxiety symptoms can benefit from reducing their intake of stimulants such as coffee, tea, cola and energy drinks as these can exacerbate symptoms 135. Partners should be aware that consuming alcohol during pregnancy is dangerous to the developing baby 136. Partners should be aware that alcohol and drugs make you feel better for a short time only 137. Partners should be aware that managing stress and worry with use of alcohol and drugs is likely to cause additional problems 138. Partners should seek professional help if they need support to manage their alcohol or drug use 139. Partners should be aware that acceptance creates a healthier, happier, and more positive environment for the whole family 140. Partners should be aware that acceptance is an attitude that can reduce the stress and challenges of working together to raise children 141. Partners should be aware that no matter how bad things get, they are temporary, e.g., babies will begin to sleep more, eat less often and it will get easier to take them out of the house 142. Partners should try to enjoy their family rather than feel that they are missing out on the old days 143. Partners should be willing to continually explore and adapt, as what works one day may not work the next 144. Partners should be aware that because the birth of a baby is highly anticipated, and expected to be a happy time, it is often difficult to recognise depression and anxiety symptoms 145. Partners should be aware that it’s normal to worry when they are pregnant or have become a parent but there is an important distinction between worry and extreme anxiety 146. Partners should encourage their partner to seek help if they believe they are experiencing anxiety symptoms, as seeking help early will contribute to a quicker recovery 147. Partners should be aware that untreated anxiety can impact on the pregnancy and the baby 148. Partners should be aware depression and anxiety symptoms often happen at the same time 149. Partners should be aware that their partner may have worries that they do not think are justified, but are nonetheless very real to their partner 150. Partners should avoid responding to their partner’s fears with shock or amazement 151. If their partner is experiencing problems with / anxiety, partners should:-Help them by breaking down tasks into small steps, so that even though they feel it is a challenge, they are confident they can do it 152. If their partner is experiencing problems with / anxiety, partners should:-Help them by not overly accommodating the anxiety, e.g., by not following unreasonable rules such as changing all clothing when entering the house 153. If partners notice their partner is looking unhappy or displaying negative feelings, they should approach the topic in a caring and non-judgmental way, e.g., “I’ve been noticing that you seem really down a lot lately, how are you feeling about things/yourself/the baby/motherhood?” 154. Partners should use follow-up questions to determine how their partner is feeling, e.g., “How are you doing? And if they say something like “I’m tired but I’m fine” asking, “But how are you really feeling?” 155. Partners should be aware that fathers are more likely to hide their depression 156. Partners should be aware that depression is not voluntary and isn't something you can just "snap out of" 157. Partners should encourage their partner to seek professional help if they think she or he is experiencing depression as this will benefit their health, the healthy development of their baby, and their relationship 158. Partners should offer to accompany their partner to the GP or maternal child health nurse if they are concerned that they may be experiencing depression 159. Partners should be aware that it is common for people with depression not to recognise that they need help or support, so they may reject offers of help 160. Partners should be aware that their partner may avoid help seeking because they are embarrassed or ashamed that they are experiencing depression 161. Partners should be aware that there are support groups available for families affected by postpartum depression and anxiety 162. Partners should take their partner seriously if she or he talks about not wanting to live or about harming themselves, e.g., by letting their partner know that they understand their feelings are real to them, no matter how bad or unreasonable they sound 163. Partners should seek professional help immediately if their partner is having thoughts of suicide or harming themselves or the baby, e.g., go to the local emergency room 164. Partners should risk conflict with their partner in the short term by getting help for them if they are concerned about their mental health, particularly if the wellbeing of the baby is at risk 165. If the mother is admitted to a mother-baby unit, partners should make the most of visiting times to maintain contact with her and the baby 166. Partners should seek professional help for their partner if they are acting in an unusual or bizarre way, e.g., being extremely withdrawn or fearful, or hearing or seeing things that others can’t | 1. Partners should be aware that having a baby may strengthen their relationship 2. Partners should be aware that when they become new parents, they will lose a bit of their identity and what their relationship used to be 3. Partners should be aware that the transition to parenthood is one of the most important times to build their relationship 4. Partners should be aware that if their partner is depressed they are at increased risk of also becoming depressed 5. Partners should acknowledge how physically uncomfortable pregnancy is for the child-bearing mother 6. Partners should be aware that hormonal changes will affect the child-bearing mother’s mood and energy levels 7. Both mothers and fathers should attend antenatal classes 8. If health professionals unknowingly overlook the father, the couple should speak to the person about making changes to involve the father more 9. Partners should help the child-bearing mother with heavy lifting and carrying as much as possible 10. Partners should discuss the possibility of the new mother and baby needing medical attention following birth ahead of time 11. Partners should let each other talk about their respective experiences of childbirth 12. Partners should seek counselling if they experience a traumatic birth 13. Partners should let each other cry or be emotional without necessarily trying to fix the problem 14. Partners should be prepared to listen even if they feel that they are hearing the same things over and over 15. Partners should avoid asking too many questions when listening, as this can sound like an interrogation 16. Partners should set aside time every day to talk without distraction 17. Partners should use open-ended questions (questions that encourage someone to talk instead of giving a yes or no answer), e.g., “How are you finding being a parent?” 18. Partners should use tentative words such as “might”, “could be” and “I wonder if” when offering advice 19. Partners should be aware that suppressing their thoughts and feelings can cause their partner to feel shut out 20. Partners should avoid telling each other how they should feel 21. Partners should be aware that conflict is a natural part of relationships 22. If their partner says or does something hurtful, the partner should look for positive or neutral intentions behind it, e.g., “I know that you’re working long hours because you want to take care of us" 23. Partners should avoid bringing up the past during conflict, e.g., “This is just like last time!" 24. Partners should avoid raising conflict topics in front of other people 25. Partners should agree that either person can raise a problem for discussion at any time 26. Partners should agree to raise problems at a good time and place, e.g., when there are no other competing demands such as dinnertime 27. Partners should engage in problem solving together by identifying the problem, brainstorming solutions, choosing a solution, evaluating the solution, and making a follow-up action plan 28. When problem solving, partners should:-Avoid accepting a situation that one partner isn’t happy with 29. Partners should avoid discussing the division of labour when they are angry or upset 30. Partners should divide the labour so that both parents have quality time and contact with each other and the baby 31. Partners should share child-care responsibilities from the beginning 32. Partners should arrange to both be at home for at least the ﬁrst week or two after the birth 33. Partners should help with the cleaning 34. Partners should help with housework before having to be asked by the primary caregiver 35. Partners should help the primary caregiver with preparing meals, e.g., food shopping, cooking, clearing the table 36. Partners should be aware that fathers may feel less skilled in handling the baby 37. Fathers should talk and share experiences with other fathers 38. Partners should seek / and accept support from:-Family 39. Partners should challenge negative thinking by pointing out situations or tasks that their partner has handled well 40. Partners should acknowledge their togetherness by saying something such as “It’s so good we’ve been through this together” 41. Partners should set aside quiet time to spend together while the baby is sleeping, even if it is only for 10 minutes 42. Partners should try to get outdoors together with the baby as much as possible 43. If their partner is resistant to going out, partners should think of things that they can do together in the home that give them a break from parenting, e.g., board games, watching a movie 44. Partners should be aware that one of the greatest causes of unhappiness and conﬂict for fathers is a lack of physical intimacy with their partner during the pregnancy and following childbirth 45. Partners should reassure each other that it is OK if they are not interested in sex 46. If partners need help with their sexual relationship they should talk to a professional, e.g., a relationship counsellor 47. Partners should encourage each other to stay hydrated 48. Partners should eat regularly throughout the day, because low blood sugar results in low mood and frustration 49. Partners should be aware that alcohol is a depressant 50. Partners should be aware that alcohol and drugs are bad for your physical health 51. Partners should avoid using alcohol or drugs 52. Partners should monitor each other for withdrawal or change in mood 53. If their partner is experiencing depression, partners should also seek professional help for themselves 54. Partners should be aware that depression is temporary and their partner will recover 55. Partners should be aware that pushing too hard for someone with depression to seek treatment may make them feel undermined and thus reject assistance outright 56. If partners are concerned that their partner is experiencing depression, they should talk about the positive effects of getting help – both for the person and the rest of the family - by printing information from a quality website to leave with them to read and think about 57. Partners should go to the doctor themselves for information and advice about depression, if their partner initially refuses to go | 1. Partners should discuss what they have lost in becoming parents and see what the similarities and differences are 2. Partners should consider seeking relationship counselling to help them identify their concerns and expectations about parenthood 3. Partners should attend antenatal appointments together 4. Partners should be aware that attending appointments together will help them develop the feeling that they are becoming parents together 5. Partners should be aware that there is very little they can do to help the child-bearing mother during labour 6. Partners should actively listen to help each other feel / acknowledged and supported by:-Restating their partner’s comments in their own words to confirm that they understand 7. Partners should use a phrase such as a 'red day' to let each other know they have had a bad day, as this may be easier to say 8. Partners should avoid expressing anger and resentment toward each other 9. Partners should avoid getting home from work late in order to avoid arguments 10. Partners should weigh up whether an issue is really important before raising it 11. Partners should use questions that begin with “Why” or “How”, or “What do you think about …?” to encourage creative thinking and problem-solving skills 12. Partners should hire a house cleaner, if affordable 13. Partners should help with the shopping 14. If the baby is unsettled, partners should take the baby for walks in the pram 15. Partners who are working should let the primary caregiver sleep in on the weekends 16. Partners should seek / and accept support from:-Friends 17. Partners should seek / and accept support from:-Parent groups 18. Partners should seek / and accept support from:-Online forums 19. Partners should seek / and accept support from:-Support groups 20. Partners should seek / and accept support from:-Play groups 21. Partners should seek / and accept support from:-Workmates 22. Partners should be aware that although they may have looked toward bosses, co-workers or friends to feel good about themselves before the baby came, they will now be each other’s main source of self-esteem 23. Partners who are working should telephone their partner from work, or drop in for lunch occasionally if they work close to home 24. Partners should show interest in their partner’s activities by asking about them and joining in when invited, e.g., taking an evening class together 25. Partners should ask a friend or family member to take the children for a couple of hours each week so that they that they can have time alone or with each other 26. If their partner is experiencing problems with / anxiety, partners should:-Help them face anxiety-provoking situations, whilst acknowledging that this is difficult 27. If their partner is experiencing problems with / anxiety, partners should:-Encourage them to consider taking supplements such as magnesium and calcium, as these are effective in reducing anxiety 28. Partners should seek relationship counselling during pregnancy if they have a history of mood problems 29. If their partner is uncomfortable expressing vulnerable emotions, partners should use rating scales to gauge their mood, e.g., “On a scale of 1-10, how agitated/tired/sad do you feel?” |
| Round 2 | 1. Partners should be aware that if their partner is depressed they are at increased risk of also becoming depressed 2. Parents should discuss their parenting hopes, fears, and roles following the birth of their infant* 3. Partners should review their priorities and expectations following the birth of their infant* 4. Partners should be aware that learning about the changes and difficulties that come with being a new parent will not protect them against experiencing these difficulties, but it may help them to recognise and respond to them, if they occur* 5. Partners should be aware that they may experience difficulties during the transition to parenthood, regardless of whether or not they have a supportive relationship* 6. Partners should be aware that each parent may be vulnerable and competent during different developmental stages of their child's life* 7. Partners should be aware that hormonal changes will affect the child-bearing mother’s mood and energy levels 8. Partners should let each other talk about their respective experiences of childbirth 9. Partners should seek professional help if they experience a traumatic birth and do not feel they are coping well afterwards* 10. The child-bearing mother should be aware that their partner may have strong feelings about the birth and need to debrief* 11. Partners should ask the child bearing mother what level of support they would like during the childbirth but be prepared to be flexible on the day* 12. Partners should be aware that they can provide the child-bearing mother with support during labour (e.g., massage, acupressure, reassurance, verbal encouragement)* 13. Partners should be prepared to listen even if they feel that they are hearing the same things over and over 14. Partners should be aware that suppressing their thoughts and feelings can cause their partner to feel shut out 15. Partners should be aware that conflict is a natural part of relationships 16. Partners should agree to raise problems at a good time and place, e.g., when there are no other competing demands such as dinnertime 17. Partners should engage in problem solving together by identifying the problem, brainstorming solutions, choosing a solution, evaluating the solution, and making a follow-up action plan 18. When problem solving, partners should take responsibility for their own behaviour and the impact of it* 19. Partners should arrange to both be at home for at least the ﬁrst week or two after the birth 20. Partners should discuss any differences in parenting to ensure that both parents are happy with how the infant is being parented* 21. Partners should discuss how the primary caregiver will be supported with childcare and home duties if their partner is unable to assist (e.g., hire a cleaner)* 22. Partners should be aware of pressure from others (e.g., parents, in-laws, colleagues)* 23. Partners should trust their own knowledge and understanding of the baby* 24. Partners should seek and accept support from whomever they both feel comfortable inviting into their home or helping with their child* 25. Partners should discuss and consider what supports they will draw on when they become parents* 26. Partners should provide practical support so that the primary caregiver can focus on resting and feeding the baby until the primary caregiver feels recovered or able to take on more duties* 27. Partners should consider having a word/phrase/excuse to use if visitors are becoming overwhelming* 28. Partners should consider seeking and accepting support from the following sources, if appropriate to their circumstances: Family* 29. Partners should be aware that their partner's attitude towards their parenting affects how confident they feel in caring for the infant* 30. Partners should set aside quiet time to spend together while the baby is sleeping, even if it is only for 10 minutes 31. Partners should try to get outdoors together with the baby as much as possible 32. If their partner is resistant to going out, partners should think of things that they can do together in the home that give them a break from parenting, e.g., board games, watching a movie 33. Partners should reassure each other that it is OK if they are not interested in sex 34. Partners should be aware that pregnancy, birth, and parenting can affect sexual health and intimacy* 35. Partners should be aware that even if the birth wasn't traumatic, it may impact their sexual relationship* 36. Partners should be aware that their sexual relationship following the birth of their infant may be different to "pre-baby" but not necessarily worse* 37. If the mother is breastfeeding, their partner should support them by making sure they are hydrated and getting enough sleep* 38. Partners should be aware that having a healthy diet will help them cope with less sleep and recovering from birth or breast-feeding* 39. Partners should be aware that there are healthier ways of coping than alcohol or drug use* 40. Partners should be aware that a partner’s use of alcohol or drugs following childbirth is associated with increased risk of perinatal depression and anxiety* 41. Partners should monitor each other for withdrawal or change in mood 42. Partners should go to the doctor themselves for information and advice about depression, if their partner initially refuses to go 43. Partners should be aware that it is often difficult to recognise depression and anxiety symptoms* 44. If partners can have agreed strategies for seeking help for different difficulties with being a new parent (e.g., We will call his mother if we need help with cleaning the house, we will go to the MCH nurse if we need help with sleeping, we will go to our friend's house for a sleep over if we need to catch up on our sleep) this can assist the family to seek help if things get tough* 45. Partners should be aware that their partner may avoid help seeking because they do not want to acknowledge that they are not coping* 46. Partners should be aware that their partner may avoid help seeking because they do not want to acknowledge that their illness may be harmful to their baby* 47. Partners should be aware that their partner may avoid help seeking because of a wide range of worries or concerns* | 1. Partners should be aware that not all parents will experience difficulties with the transition to parenthood* 2. Partners should talk about their own feelings, assumptions, or stigmas relating to mental illness* 3. Partners should say something straight away if they are having problems with their midwife* 4. Partners should be aware of the difference between being critical and attacking, i.e., criticism can be given and heard as well-meaning and constructive, while attacking is hurtful* 5. Partners should be aware that some conflict is inevitable and may never be properly resolved* 6. Partners should arrange to both be at home for at least the ﬁrst week or two after the birth, if this is feasible given their financial arrangements* 7. Partners who are in paid work should be aware that sometimes nothing will get done while they are at work* 8. Partners should be aware that stimulants such as coffee, tea, cola, and energy drinks can exacerbate anxiety symptoms* 9. Partners should eat quick and easy meal options that incorporate healthy foods* | 1. Partners should be aware that having a baby may strengthen their relationship 2. Partners should be aware that when they become new parents, they will lose a bit of their identity and what their relationship used to be 3. Partners should be aware that the transition to parenthood is one of the most important times to build their relationship 4. Partners should consider seeking relationship counselling to help them identify their concerns and expectations about parenthood, if they are having trouble negotiating these concerns among themselves* 5. Partners should consider engaging in counselling during pregnancy as a means of preparing to become a family* 6. Partners should be aware that if their relationship is not as supportive as they would like, having a baby can make you work better together as a couple* 7. Partners should try to develop an understanding of their extended family's background and attitudes towards mental illness* 8. Partners should acknowledge how physically uncomfortable pregnancy is for the child-bearing mother 9. Both mothers and fathers should attend antenatal classes 10. If health professionals unknowingly overlook the father, the couple should speak to the person about making changes to involve the father more 11. Partners should help the child-bearing mother with heavy lifting and carrying as much as possible 12. Partners should discuss the possibility of the new mother and baby needing medical attention following birth ahead of time 13. Partners should seek counselling if they experience a traumatic birth 14. Partners should be aware that talking about the birth without the support of a skilled third party can result in further traumatizing or re-triggering* 15. Partners should let each other cry or be emotional without necessarily trying to fix the problem 16. Partners should avoid asking too many questions when listening, as this can sound like an interrogation 17. Partners should set aside time every day to talk without distraction 18. Partners should use open-ended questions (questions that encourage someone to talk instead of giving a yes or no answer), e.g., “How are you finding being a parent?” 19. Partners should use tentative words such as “might”, “could be” and “I wonder if” when offering advice 20. Partners should avoid telling each other how they should feel 21. If their partner says or does something hurtful, the partner should look for positive or neutral intentions behind it, e.g., “I know that you’re working long hours because you want to take care of us" 22. Partners should avoid bringing up the past during conflict, e.g., “This is just like last time!" 23. Partners should avoid raising conflict topics in front of other people 24. Partners should agree that either person can raise a problem for discussion at any time 25. When problem solving, partners should avoid accepting a situation that one partner isn’t happy with 26. When discussing things that might need to change, partners should use the "feedback sandwich" method (a feedback sandwich consists of criticism sandwiched between two positive comments). E.g., "Thanks for helping me out with the laundry. It would be great if you could fold the towels before putting them away. I really appreciate that you always put the washing on the line"* 27. Partners should avoid expressing resentment toward each other* 28. Partners should avoid expressing anger toward each other* 29. Partners should avoid discussing the division of labour when they are angry or upset 30. Partners should divide the labour so that both parents have quality time and contact with each other and the baby 31. Partners should share child-care responsibilities from the beginning 32. Partners should help with the cleaning 33. Partners should help with housework before having to be asked by the primary caregiver 34. Partners should help the primary caregiver with preparing meals, e.g., food shopping, cooking, clearing the table 35. Partners should be aware that fathers may feel less skilled in handling the baby 36. Fathers should talk and share experiences with other fathers 37. Partners should seek / and accept support from family 38. Partners should share the household chores (e.g., shopping and cleaning)* 39. Partners should consider seeking and accepting support from the following sources, if appropriate to their circumstances: Friends* 40. Partners should consider seeking and accepting support from the following sources, if appropriate to their circumstances: Parent groups* 41. Partners should consider seeking and accepting support from the following sources, if appropriate to their circumstances: Online forums* 42. Partners should consider seeking and accepting support from the following sources, if appropriate to their circumstances: Support groups* 43. Partners should consider seeking and accepting support from the following sources, if appropriate to their circumstances: Play groups* 44. Partners should consider seeking and accepting support from the following sources, if appropriate to their circumstances: Workmates* 45. Partners should challenge negative thinking by pointing out situations or tasks that their partner has handled well 46. Partners should acknowledge their togetherness by saying something such as “It’s so good we’ve been through this together” 47. Partners should be aware that one of the greatest causes of unhappiness and conﬂict for fathers is a lack of physical intimacy with their partner during the pregnancy and following childbirth 48. If partners need help with their sexual relationship they should talk to a professional, e.g., a relationship counsellor 49. Partners should be aware that many parents do not experience sexual problems during the transition to parenthood* 50. If their partner does not want to have sex, partners should not ask why* 51. Partners should encourage each other to stay hydrated 52. Partners should eat regularly throughout the day, because low blood sugar results in low mood and frustration 53. Partners should be aware that alcohol is a depressant 54. Partners should be aware that alcohol and drugs are bad for your physical health 55. Partners should avoid using alcohol or drugs 56. Partners should not be punitive about each other's drug or alcohol use* 57. If their partner is experiencing depression, partners should also seek professional help for themselves 58. Partners should be aware that depression is temporary and their partner will recover 59. Partners should be aware that pushing too hard for someone with depression to seek treatment may make them feel undermined and thus reject assistance outright 60. If partners are concerned that their partner is experiencing depression, they should talk about the positive effects of getting help – both for the person and the rest of the family - by printing information from a quality website to leave with them to read and think about |
| Round 3 | 1. Partners should be aware of the difference between being critical and attacking, i.e., criticism can be given and heard as well-meaning and constructive, while attacking is hurtful* | - | 1. Partners should be aware that not all parents will experience difficulties with the transition to parenthood* 2. Partners should talk about their own feelings, assumptions, or stigmas relating to mental illness* 3. Partners should say something straight away if they are having problems with their midwife* 4. Partners should be aware that some conflict is inevitable and may never be properly resolved* 5. Partners should arrange to both be at home for at least the ﬁrst week or two after the birth, if this is feasible given their financial arrangements* 6. Partners who are in paid work should be aware that sometimes nothing will get done while they are at work* 7. Partners should be aware that stimulants such as coffee, tea, cola, and energy drinks can exacerbate anxiety symptoms* 8. Partners should eat quick and easy meal options that incorporate healthy foods* |

*Items based on panel members’ feedback on Round 1.
