# Supplementary material for: Enhancing reciprocal partner support to prevent perinatal depression and anxiety: a Delphi consensus study
Source: BMC Psychiatry. 2016 Feb 3;16:23. doi: 10.1186/s12888-016-0721-0 (PMC4739319; doi:10.1186/s12888-016-0721-0)

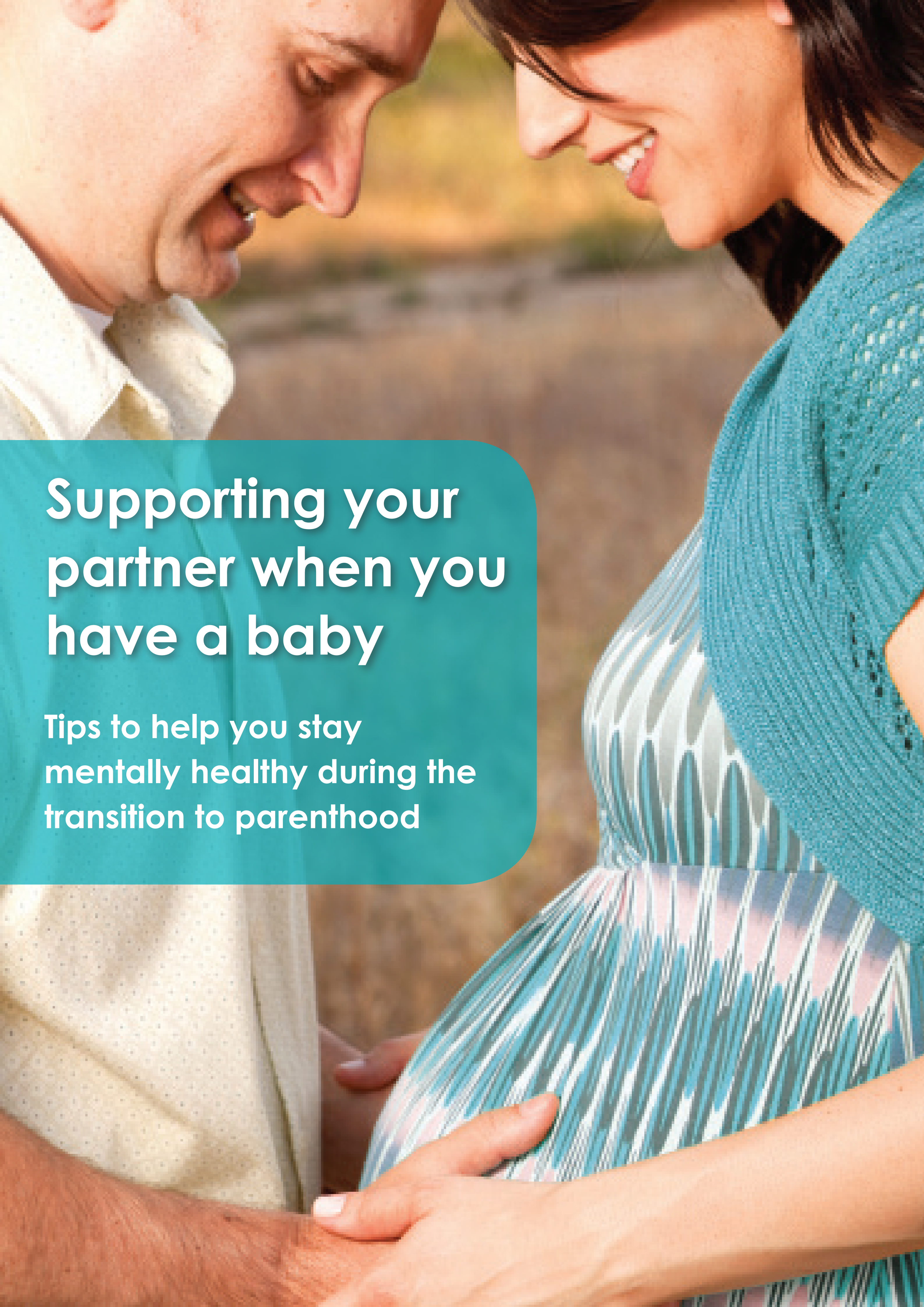

Supporting your partner when you have a baby

Tips to help you stay mentally healthy during the transition to parenthood

If you and your partner are expecting a baby, or have recently had a baby, this guide was designed for you. It provides information and suggestions on how you can support your partner during pregnancy and following childbirth to reduce the chance that he or she will experience depression or anxiety.

These recommendations are based on the expert opinions of clinicians and researchers who specialise in perinatal mental health, as well as people who have experienced perinatal depression and anxiety first hand.

The guidelines are designed to be inclusive of all couples, including partners in same-sex relationships. We recognise that each family is unique so not all of the suggestions may be relevant to you. Please feel free to adapt the guidelines to your specific situation.

Becoming parents

Most couples find the transition to parenthood challenging. It is normal to experience a wide range of emotions during pregnancy and over the following year. Even when things are progressing normally, pregnancy and new parenthood can be stressful for you, your partner, and your immediate family. Having a baby can cause changes which involve a degree of loss and grief, such as the loss of freedom, work identity, financial independence and social contacts. Caring for a baby can also place stress on your relationship with your partner. When a baby arrives, the focus shifts from self and partner-care to mostly baby-care.

What are your hopes, fears, and expectations?

Learning about the changes and difficulties that come with being a new parent will not necessarily protect you against experiencing these difficulties, but it may help you to recognise and respond to them if they occur. Before your baby is born, try to identify potential sources of stress, such as relationship problems or financial difficulties, and explore ways of dealing with these problems with your partner. Discuss your parenting hopes, fears, and roles together. Try to identify what's important to each of you - e.g., communicating with others, affection, fun, safety, financial security, time together as a family and so on - and discuss how you can meet these needs. Think about the sort of partner and parent you want to be and work out how you can achieve this. Talk about your expectations about parenting and try to ensure that your expectations are compatible and realistic. Be aware of unrealistic expectations that could cause rifts in your relationship, e.g., "I'll have the same time available for work or leisure activities as before the baby was born".

Thinking back to your own childhoods

You and your partner bring separate past experiences, ideas and hopes to your new family, and combining these can be difficult. Think back to your own childhoods and discuss how this might influence what you do and say as parents. For example, if your partner was not parented warmly themselves, they may need encouragement to spend time with your baby.

Adapting to parenthood

Following the birth of your baby it may be helpful to review your priorities and expectations with your partner. Find time to revisit your parenting hopes, fears, and roles. Be willing to continually explore and adapt, as what works one day may not work the next. You may be vulnerable or competent during different stages of your child's life. Try to enjoy your family rather than feel like you are missing out on the old days. Remember, no matter how difficult things get, the situation is temporary. Your baby will begin to sleep more, eat less often and it will get easier to take him or her out of the house.

It can be helpful, at this time, to develop an attitude of acceptance. Acceptance refers to opening up and making room for difficult feelings, sensations, and experiences. Acceptance creates a healthier, happier, and more positive environment for the whole family and can reduce the stress and challenges of working together to raise children.

> " It is normal to experience a wide range of emotions during pregnancy and the following year "

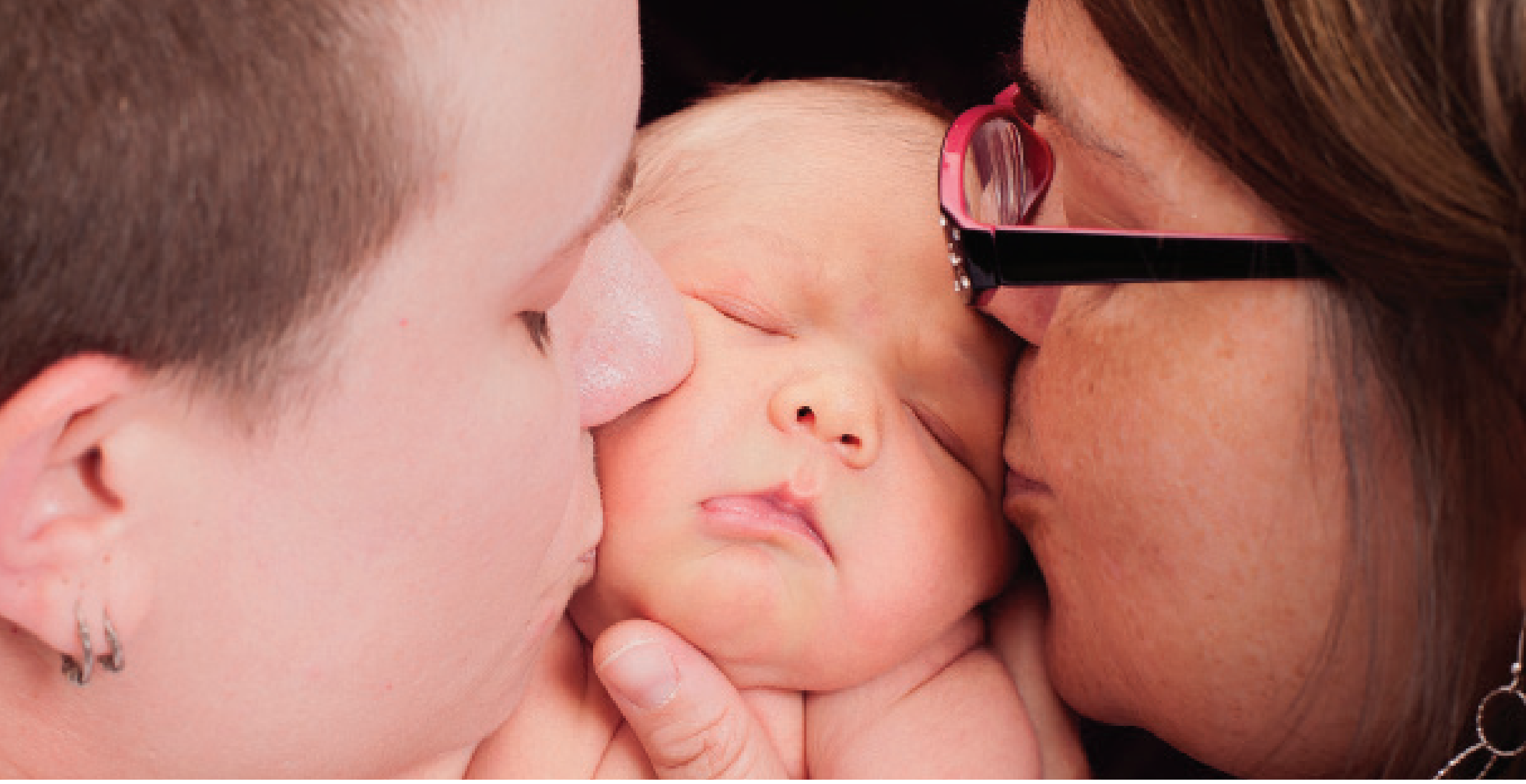

Pregnancy and childbirth

Pregnancy is a powerful and life-changing experience for mothers and fathers. Encourage your partner to rest, particularly if she is having a difficult pregnancy.

It is a good idea to discuss your birth plan and intentions for labour in advance. Share with your partner how you are feeling about labour and childbirth during pregnancy. If you are the child-bearing mother, talk to your partner about whether or not you want him or her to be present during the labour.

Do not assume that because childbirth is a natural process, the child-bearing mother will be able to cope without support. Childbirth can be anxiety-provoking. Partners can provide support during labour through massage or acupressure, and by providing reassurance and verbal encouragement. Discuss what level of support you would like in advance but be prepared to be flexible on the day.

The importance of de-briefing after birth

Mothers' and fathers' experiences around pregnancy, labour, childbirth, and early parenthood can be very different. Hormonal changes will affect the child-bearing mother's mood and energy levels and some degree of stress and fatigue is normal following childbirth. You and your partner may have strong feelings about the birth and need to debrief. Keep in mind that unlike the child-bearing mother, partners do not go through all the physical changes of pregnancy and giving birth, so they may not begin to adjust to parenthood until the baby is born. Talk to your partner about your respective experiences of childbirth.

Be mindful that if the birth was traumatic, it may impact on your sexual relationship. It may also increase your risk of developing depression and anxiety. If you experience a traumatic birth and do not feel you are coping afterward, consider seeking professional help.

Sex and intimacy

It is important to understand that even if the birth wasn't traumatic, it may impact upon your sexual relationship. Your sex life is likely to change during pregnancy and following childbirth, and may not return to normal for a year or more. Your sexual health and intimacy may be affected by a number of factors including the physical recovery, lifestyle changes after the birth, and changes in body image. Many child-bearing mothers will have less interest in sex in the later stages of pregnancy and the months following birth due to hormonal changes and the way they feel about their body.

You may feel rejected or unwanted if your partner is not interested in sex. Less interest in sex does not mean that your partner is no longer interested in you or attracted to you. Explore different types of intimacy, such as cuddling or hand holding. Your sexual relationship may be different to "pre-baby", but not necessarily worse. Reassure your partner that it is OK if they are not interested in sex. Communicate with your partner what you want and how you feel about sex.

It's also important to be aware that if your partner is feeling low and has lost interest in sex, this can be a sign of depression.

> "Explore different types of intimacy, such as cuddling or hand holding"

Showing affection and acceptance

Looking after each other enables you to care for your baby. Do what you can to strengthen your connection with your partner during pregnancy and following childbirth. Let your partner know that you love them. Do things to show your love and appreciation, such as buying flowers, making a cup of tea, or giving your partner a massage.

Making time for each other

Following childbirth it will be important to set aside quality time with your partner, whereas before you may have taken such occasions for granted. Arrange to do enjoyable activities with your partner. Set aside quiet-time to spend together while your baby is sleeping, even if it is only for 10 minutes. Think about the things you used to like doing before you had your baby and consider how you might do those activities together again. Try to get outdoors together with your baby as much as possible. If your partner is resistant to going out, think of things that you can do together in the home that give you a break from parenting, such as board games or watching a movie.

Showing your appreciation

Let your partner know that you're there for them and try to be patient and understanding. Your partner's self-esteem may be more fragile after your baby is born. Praise your partner's parenting efforts by giving specific examples, e.g., "I love how you smile at our baby". Validate your partner's thoughts, experiences, and worries, e.g., "I can see how hard this is for you", "This would be a hard time for anyone", "You have been dealing with so much lately". Let your partner know they are not failing your baby, or you, if they feel stressed. Also let your partner know if you feel you need more acknowledgment, appreciation and encouragement from them.

" Do things to show your love and appreciation, such as buying flowers, making a cup of tea, or giving your partner a massage "

Tips for communicating

The art of listening

Babies develop well if their parents can relate to each other with respect and affection. Talking is a major part of resolving problems or conflict and open communication will strengthen your relationship. Share your concerns, thoughts, and feelings with your partner. Ask your partner about their day and how they are feeling, rather than just focusing on your baby, e.g., "What was good about today?" "What wasn't so good?" Listen to your partner's concerns, even if you feel that you are hearing the same things over and over. Ask them to explain or give more information if you don't understand what's being said. When your partner wants to talk, they're not necessarily seeking advice, but may just need to talk it through. Avoid jumping to conclusions. Actively listen to help one another feel acknowledged and supported by:

- stopping what you're doing to show that you are giving your full attention
- using body language to show that you are listening, e.g., maintaining eye contact and sitting in a relaxed position
- waiting until your partner has finished speaking before offering your opinion or suggestions.

Talking about tricky topics and painful feelings

It can be difficult to find the words to talk about painful and negative thoughts. Encourage your partner to talk honestly about whatever they are struggling with. Be careful not to dismiss their concerns when providing reassurance, e.g., rather than saying something isn't a problem say "I will be here to help you with that" or "I can see that is really worrying you but I think we can get through that". You may not want to 'burden' each other with your feelings. Do not bottle-up your feelings as this makes it more likely they'll come out the wrong way, such as during an argument. Voice your needs directly rather than thinking that your partner can read your mind. Hiding your thoughts and feelings can cause your partner to feel shut out.

Professional assistance is available if you need help with communicating effectively (e.g., a couples therapist or a psychologist).

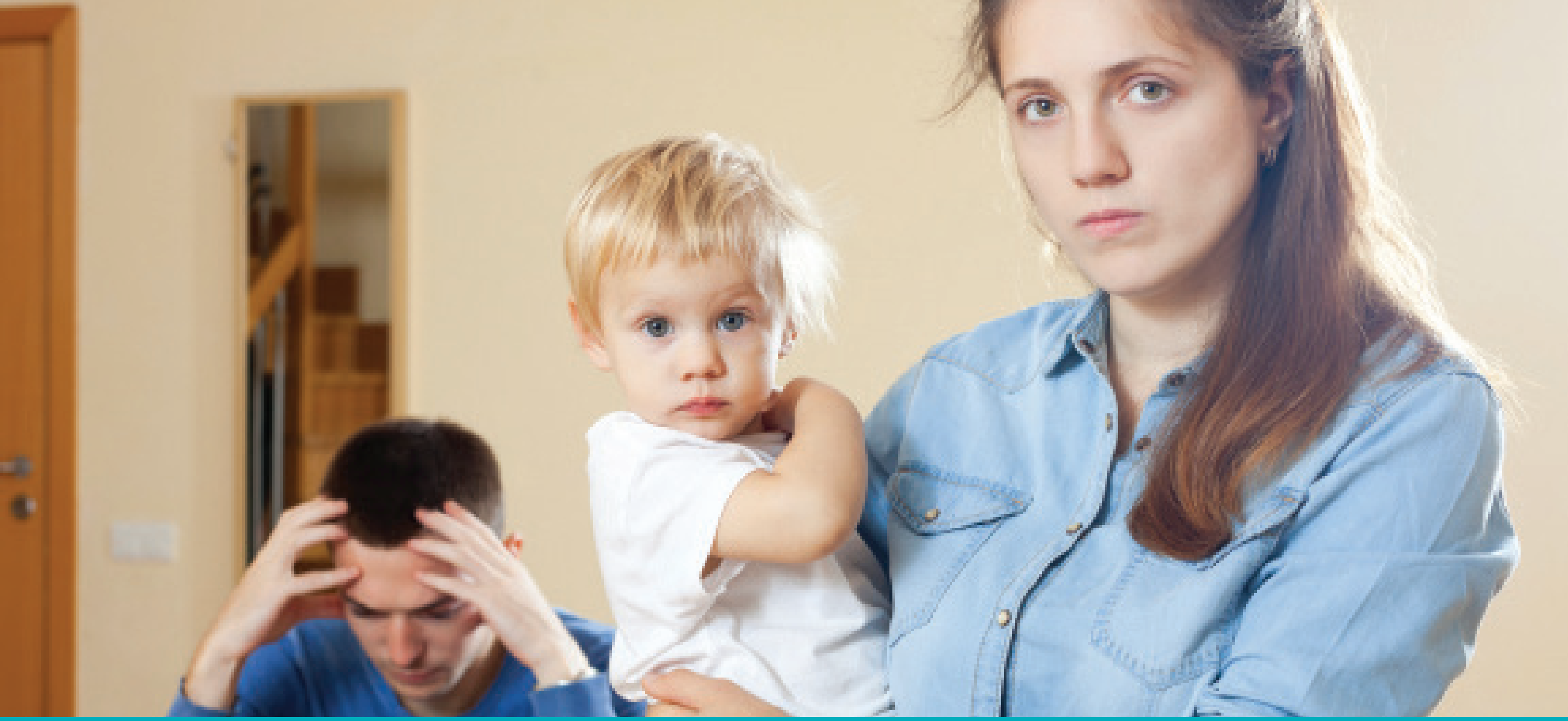

Managing conflict

Conflict is a natural part of relationships. It is normal for couples to experience an increase in arguments and tension during pregnancy and following childbirth. Managing conflict well will benefit your child/children. You can't always pre-empt or fix everything that goes wrong. Try to resolve small conflicts before they escalate into major rifts.

How to express your concerns respectfully

Agree to raise problems at a good time and place, when there are no other competing demands, e.g., when your children are in bed. If your partner doesn't want to talk about the issue at the time it is first raised, schedule another time to discuss it, but avoid letting fights continue overnight.

Try to express your needs without criticising your partner. Be mindful of what you say, and how you say it, as it may influence how your partner responds. Try to use 'I' statements, e.g., Instead of saying, "You don't make any time for us anymore", say "I feel lonely when we spend less time together". Describe what's causing your concerns without saying why you think it is happening, e.g., instead of saying "You just sit down and watch TV while I have to get the dinner ready and look after the children", say "I'd find it easier to get dinner if the kids were kept busy. Would you be able to spend some time with them?" Avoid words or phrases that imply that your partner is always wrong or not trying, e.g., "You always ..." or "You never ..." Take responsibility for your own behaviour and its impact.

Problem solving as a team

Engage in problem solving with your partner by breaking things down into these steps:

1. identify the problem
2. brainstorm solutions
3. choose a solution
4. evaluate the solution
5. make a follow-up action plan

When you are problem solving together, take turns talking. Stay focused on the topic, rather than side-track the conversation by raising other issues or concerns. Try to hear the positive in what your partner is saying. Try to understand your partner's point of view even if you don't agree, e.g., "I can understand why you're angry that I asked my parents over for the weekend without talking to you first". Offer suggestions or examples rather than dictating to your partner what to do or trying to force them to change. Review your progress by discussing what worked well, what didn't work, and what you should change.

Avoiding being critical

Avoid name-calling or making unfavourable comparisons to other parents, e.g., "You're stupid!" Try not to judge your partner, e.g., thinking in terms of who is right and who is wrong, or thinking of your partner as the enemy or "the one with the problem". Avoid criticising your partner's body or demanding that they lose weight. If you feel criticised by your partner, give feedback about how you are feeling, e.g., "I feel… when you do / say..." Be aware of the difference between giving feedback and attacking, i.e., feedback can be given and heard as well-meaning and constructive, while attacking is hurtful.

It's a good idea to try to become aware of your own and your partner's warning signs that you're becoming overwhelmed, e.g., clenched jaw, raised voice, door slamming, irritability, indecisiveness. Take a break if your tempers are too hot and return to communicating when you are calmer, e.g., say something like, "I want to listen to you. I know this is important, but I'm having a hard time because we're so mad at each other. Can we take a break and talk about it later?"

Seek professional help if you are having difficulty resolving your relationship problems.

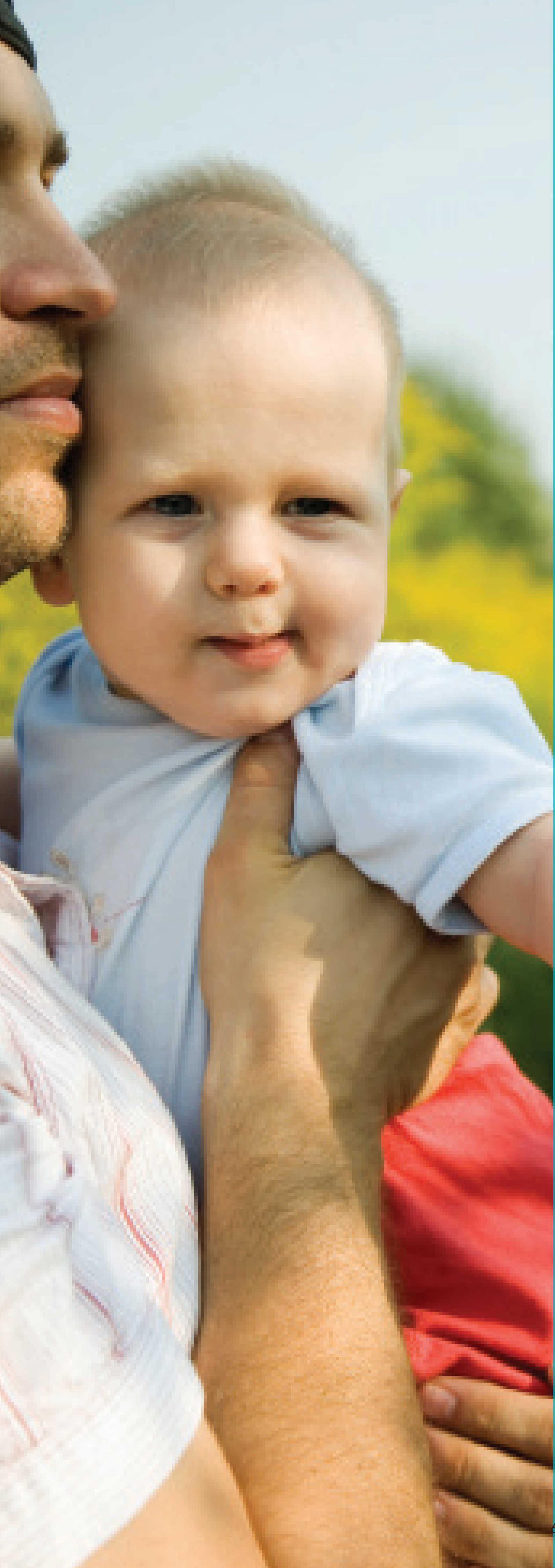

Sharing the workload

Your daily routines will change following childbirth. It can be helpful to plan the division of household labour and agree on who does what before your baby is born. Talk about who will be employed in paid work. Be willing to re-negotiate the division of labour as needed. Discuss how the primary caregiver will be supported with childcare and home duties if the other is unable to assist (e.g., hire a cleaner).

Arrange for both of you to be at home for at least the first week or two after the birth. Try to share the household chores. If you are not the primary caregiver, try to support her/him so that they can focus on resting and feeding your baby for the first six weeks, or until they feel able to take on more duties.
If you are the primary caregiver, communicate that you need help by specifically stating what you need, e.g., Instead of saying, "I feel overwhelmed and need help around here" ask, "Would you please do the laundry for me this week? I'm feeling so overwhelmed". Remember to acknowledge your partner's practical support.

Providing the primary caregiver with breaks

Try to arrange things so that the primary caregiver has some leisure time at least once a week. Provide the primary caregiver with breaks that they can count on, by doing things such as taking the baby out for a walk. Help the primary caregiver have time away from the baby doing something they find pleasurable (e.g., a massage or a warm bath). Try to help out rather than get angry if your partner is finding it hard to cope with everyday chores. Take the baby if your partner is getting upset or flustered.

Being involved in childcare

If you are the primary caregiver, encourage your partner to be involved with the baby and give them space to do this without watching over them, as this will build their confidence and help them build a strong relationship with their child. Your attitude towards your partner's parenting affects how confident they feel in caring for your baby. Accept that you may do things differently from your partner, and that these different experiences can be good for your baby. Discuss any differences in parenting to ensure that you and your partner are happy with how your baby is being parented.

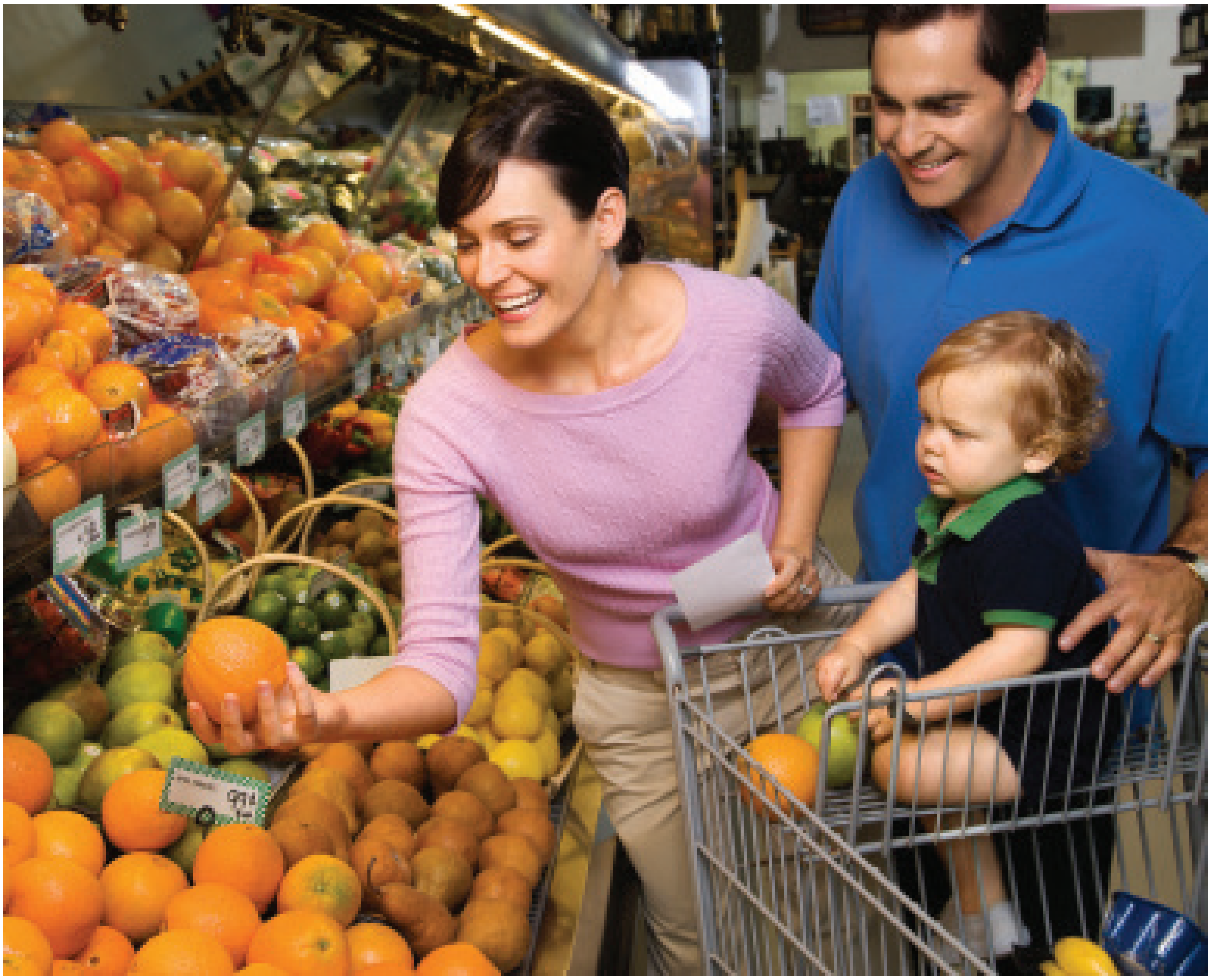

Staying healthy

Diet

Having a healthy diet will help you cope with less sleep and recover from birth or breast-feeding. Look for quick and easy meal options that incorporate lean meats, whole-grains, low-fat dairy products, fresh fruit, and vegetables. Choose healthy snacks (e.g., fruit, low-fat yoghurt, raw nuts and seeds, wholegrain crackers) over highly processed foods such as biscuits, cake, and chocolate. If you have anxiety, you may benefit from reducing your intake of stimulants such as coffee, tea, cola and energy drinks as these can exacerbate symptoms.

Sleep

Encourage your partner to sleep when needed.

Exercise

Encourage your partner to be physically active either individually or with you. Help your partner to get out and get some fresh air.

Breastfeeding

If your partner is breastfeeding, support her by making sure she is hydrated and getting enough sleep.

Alcohol and drugs

There are a lot of challenges when you become a parent, and it can be tempting to use drugs and alcohol to cope. Although alcohol and drugs make you feel better for a short time, they can also lead to additional problems. Consuming alcohol during pregnancy can be dangerous to the developing baby, while excessive use of alcohol or drugs following childbirth is associated with increased risk of depression and anxiety. If you think that you might be consuming excessive alcohol or drugs, there are healthier ways of coping.

Seeking help from family and friends

Existing supports and friendships can change when you become parents. During pregnancy, identify support people who will help you following childbirth. Discuss and consider what supports you will draw on when you become parents. If one of you works away from home, a good support network is particularly important. Where possible, have agreed strategies for seeking help for different difficulties (e.g., We will call your mother if we need help with cleaning the house, we will go to the Maternal Child Health nurse if we need help with sleeping, we will go to our friend's house for a sleep over if we need to catch up on our sleep).

Seek and accept support from whomever you and your partner feel comfortable inviting into your home or helping with your child. For example, consider seeking and accepting support from family, if appropriate to your circumstances. Be aware of the pressure and the expectations of others (e.g., parents, in-laws, family, colleagues) and trust your own knowledge and understanding of your baby. Discuss and negotiate whether extended family are being supportive or intrusive. If you are becoming overwhelmed, tactfully limit visitors and establish boundaries by making sure visitors don't outstay their welcome or turn up at inconvenient hours. Consider having a word/phrase/excuse to use if visitors are becoming overwhelming.

> “ Be aware of the pressure and the expectations of others and trust your own knowledge and understanding of your baby ”

How can I tell if my partner is experiencing perinatal depression or anxiety?

Because the birth of a baby is highly anticipated, and expected to be a happy time, it is often difficult to recognise depression and anxiety symptoms. Monitor your partner for withdrawal or change in mood.

Fathers are more likely to hide their depression. Be aware of the symptoms of depression that may be more typical of fathers. Your partner acting out of character may indicate that they need help adjusting to parenthood.

If you notice your partner is looking unhappy or displaying negative feelings, approach the topic in a caring and non-judgmental way, e.g., "I've been noticing that you seem really down a lot lately, how are you feeling about things/yourself/the baby/ parenthood?" Use follow-up questions to determine how your partner is feeling, e.g., "How are you doing? And if they say something like "I'm tired but I'm fine" ask, "But how are you really feeling?"

How you can help your partner manage their anxiety

If your partner is experiencing problems with anxiety, help them by breaking down tasks into small steps, so that even though they feel it is a challenge, they are confident they can do it. Try not to overly accommodate the anxiety. For example, try not to follow unreasonable rules such as changing all clothing when entering the house.

Your partner may have worries that you do not think are justified, but are nonetheless very real to them. Try to avoid responding to your partner's fears with shock or amazement.

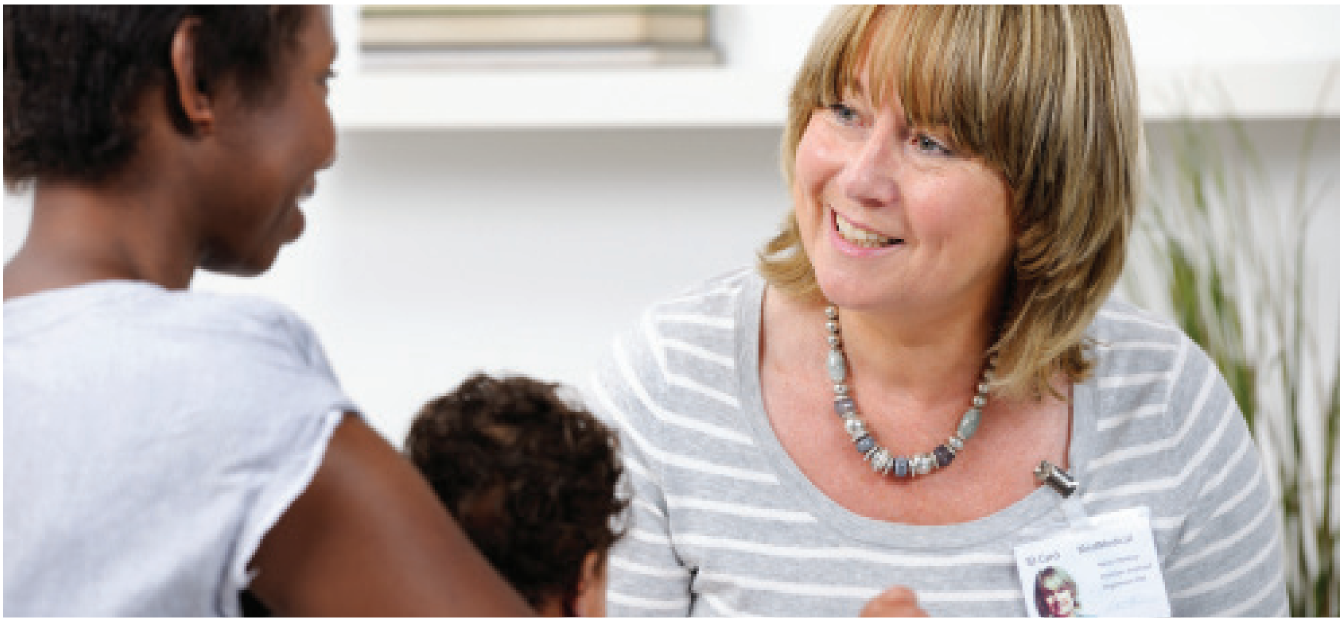

What do we mean by

perinatal depression and anxiety?

The term 'perinatal' describes pregnancy and up to one year after birth. When we talk about perinatal depression and anxiety we mean depression and anxiety disorders that develop during pregnancy or following the birth of a baby.

Perinatal depression is different from the 'baby blues'. Approximately 50 to 80% of women experience the 'baby blues' between the third and tenth day following birth. During this time women may be more tearful than usual and feel overwhelmed. These feelings usually pass within a few days. In contrast, perinatal depression persists for at least two weeks and the symptoms interfere with your ability to cope with day-to-day activities.

Perinatal anxiety refers to the presence of severe, long-lasting anxiety symptoms during pregnancy or up to one year after birth. The anxiety is strong enough to make it difficult to cope with day-to-day life and causes problems in your relationships with family and friends.

Symptoms of perinatal depression

- Low mood and/or feeling numb most of the day, nearly every day
- Feeling inadequate and/or excessive guilt
- Loss of interest in things that you would normally enjoy
- Being unable to fall asleep or get back to sleep or sleeping excessively
- Not eating or over-eating
- Feeling unmotivated and unable to cope with the daily routine
- Withdrawing from friends and family
- Not looking after yourself properly
- Decreased energy and feeling exhausted
- Having trouble concentrating, making decisions, or remembering things
- Having thoughts about harming yourself or the baby, ending your life, or wanting to escape

Source: beyondblue. (2015). Symptoms of depression. Beyondblue: Melbourne, Australia. www.beyondblue.org.au

Symptoms of perinatal anxiety

- Anxiety or fear that interrupts your thoughts and interferes with daily tasks
- Panic attacks - outbursts of extreme fear and panic that are overwhelming and feel uncontrollable
- Anxiety and persistent worries that keep coming into your mind
- Constantly feeling irritable, restless or 'on edge'
- Having tense muscles, a 'tight' chest and heart palpitations
- Finding it difficult to relax and/or taking a long time to fall asleep at night
- Anxiety or fear that stops you going out with your baby
- Anxiety or fear that leads you to check on your baby constantly
- Fear for the baby and/or fear of being alone with the baby or the baby being unsettled

Source: Adapted from beyondblue. (2015). Symptoms of anxiety. Beyondblue: Melbourne, Australia. www.beyondblue.org.au

Symptoms more typical of fathers

- Tiredness, headaches and pain
- Irritability, anxiety and anger
- Changes in appetite
- Feelings of being overwhelmed, out of control, and unable to cope
- Increased risk tasking
- Changes to sleep patterns, especially a lack of sleep
- Feeling isolated and disconnected
- Withdrawal from intimate relationships and from family, friends and community life
- Increased hours at work
- Increased alcohol or drug use

Source: Adapted from How Is Dad Going? (2015). Postnatal depression and anxiety in dads. Post and Antenatal Depression Association: Melbourne, Australia. www.howisdadgoing.org.au

Risk factors for perinatal depression and anxiety

Risk factors are characteristics or experiences that increase the likelihood that someone will develop an illness. Researchers have identified the following risk factors for perinatal depression and anxiety. If you have one or more of these risk factors it does not necessarily mean you will develop perinatal depression or anxiety – each person is unique and responds to triggers differently.

- Personal or family history of mental health problems or current mental health problems
- Pregnancy, labour or delivery complications
- Perinatal loss, e.g., miscarriage, stillbirth or termination
- Current or past history of physical, psychological, or sexual abuse
- Anxious or perfectionist personality
- Lack of support from family and friends
- Stressful life events (e.g., moving house)
- Continuing lack of sleep or rest
- Unplanned pregnancy
- Having multiples (e.g. twins or triplets)
- Severe baby blues after the birth
- Premature baby
- Difficulties with breastfeeding
- A baby that is difficult to settle
- Partner experiencing perinatal depression or anxiety

> “ Depression is not voluntary and isn’t something you can just snap out of”

Sources: Adapted from beyondblue. (2015). Life factors that increase risk. Beyondblue: Melbourne, Australia. www.beyondblue.org.au and Black Dog Institute (2013). Causes and risk factors. Black Dog Institute: Sydney, Australia www.blackdoginstitute.org.au

Seeking help

If you are concerned that your partner is experiencing depression or anxiety offer to accompany them to the GP or maternal child health nurse. Seeking professional help will benefit your health, the healthy development of your baby, and your relationship. Depression is not voluntary and isn’t something you can just “snap out of”. Untreated anxiety can impact on the pregnancy and your baby. Seeking help early will contribute to a quicker recovery.

What if my partner is reluctant to seek help?

It is common for people with depression and anxiety not to recognise that they need help or support, so they may reject offers of help. Your partner may avoid help seeking because of a wide range of worries or concerns. For example, they may not want to acknowledge that they are not coping or that their illness may be harmful to the baby. Your partner may avoid help seeking because they are embarrassed or ashamed that they are experiencing depression or anxiety. If your partner initially refuses to go to the GP, go to the doctor for information and advice by yourself. There are also support groups available for families affected by perinatal depression and anxiety.

What to do if you, your partner, or your baby is at risk of harm

Take your partner seriously if she or he talks about not wanting to live or about harming themselves. One way you can do this is by letting your partner know that you understand their feelings are real to them, no matter how bad or unreasonable they sound. Seek professional help immediately (e.g., go to your local emergency department) if:

- your partner is having thoughts of suicide or harming themselves or the baby
- your partner is acting in an unusual or bizarre way, e.g., being extremely withdrawn or fearful, or hearing or seeing things that others can’t.

If you are concerned about your partner’s mental health, risk conflict with them in the short term by getting help for them, particularly if the wellbeing of your baby is at risk. If your partner is admitted to a mother-baby unit, make the most of visiting times to maintain contact with her and the baby.

Services and support

Parenting

Services are available to help with the challenging aspects of parenting. Support is available from Maternal Child Health nurses, early parenting centres, and baby sleep clinics. Find out more about these services by contacting your local council.

Pregnancy Birth & Baby Helpline
pregnancybirthbaby.org.au
1800 882 436

Raising Children Network
raisingchildren.net.au

Parentline
parentline.com.au
1300 30 1300

Mental health support

Lifeline
lifeline.org.au
13 11 14

Post and Antenatal Depression Association Inc (PANDA)
panda.org.au
1300 726 306

Beyondblue
beyondblue.org.au
1300 22 4636

Centre of Perinatal Excellence (COPE)
cope.org.au
9376 6321

Relationships

Relationships Australia
relationships.org.au
1300 364 277

Fathers

Mens Line
mensline.org.au
1300 78 99 78

How is dad going?
howisdadgoing.org.au

Intimate partner violence

Intimate partner and family violence occurs when someone who has a close personal relationship with you makes you feel afraid, powerless or unsafe. It can be physical, but can also be emotional and psychological. If you do not feel safe and need immediate help call 000 or go to your local hospital emergency department.

National Sexual Assault, Domestic Family Violence Counselling Service
1800respect.org.au
1800 737 732

How these guidelines were developed

These guidelines were produced using the Delphi method, which is a systematic way of assessing the consensus of a panel of experts. A wide range of potential actions were derived from a review of websites and research on partner support and perinatal depression and anxiety. The actions included in the guidelines were rated as important or essential by expert panels of perinatal mental health professionals and individuals with experience of perinatal depression or anxiety, either as a consumer or a carer.

Details of the methodology can be found in Pilkington, P.D., Milne, L.C., Cairns, K.E, & Whelan, T.A. Enhancing reciprocal partner support to prevent perinatal depression and anxiety: a Delphi consensus study (in submission).

ACU AUSTRALIAN CATHOLIC UNIVERSITY

Partners to Parents

Although these guidelines are copyright, they can be freely reproduced for non-profit purposes provided the source is acknowledged.

Please cite these guidelines as follows: Pilkington, P.D., Milne, L.C., Cairns, K., & Whelan, T. (2015). Supporting your partner when you have a baby [Brochure]. Melbourne, Australia: Partners to Parents.

Enquiries should be sent to: Pam Pilkington, School of Psychology Faculty of Health Sciences,
Australian Catholic University, Locked Bag 4115, Fitzroy, VIC 3065, Australia.
Email: pam.pilkington@acu.edu.au

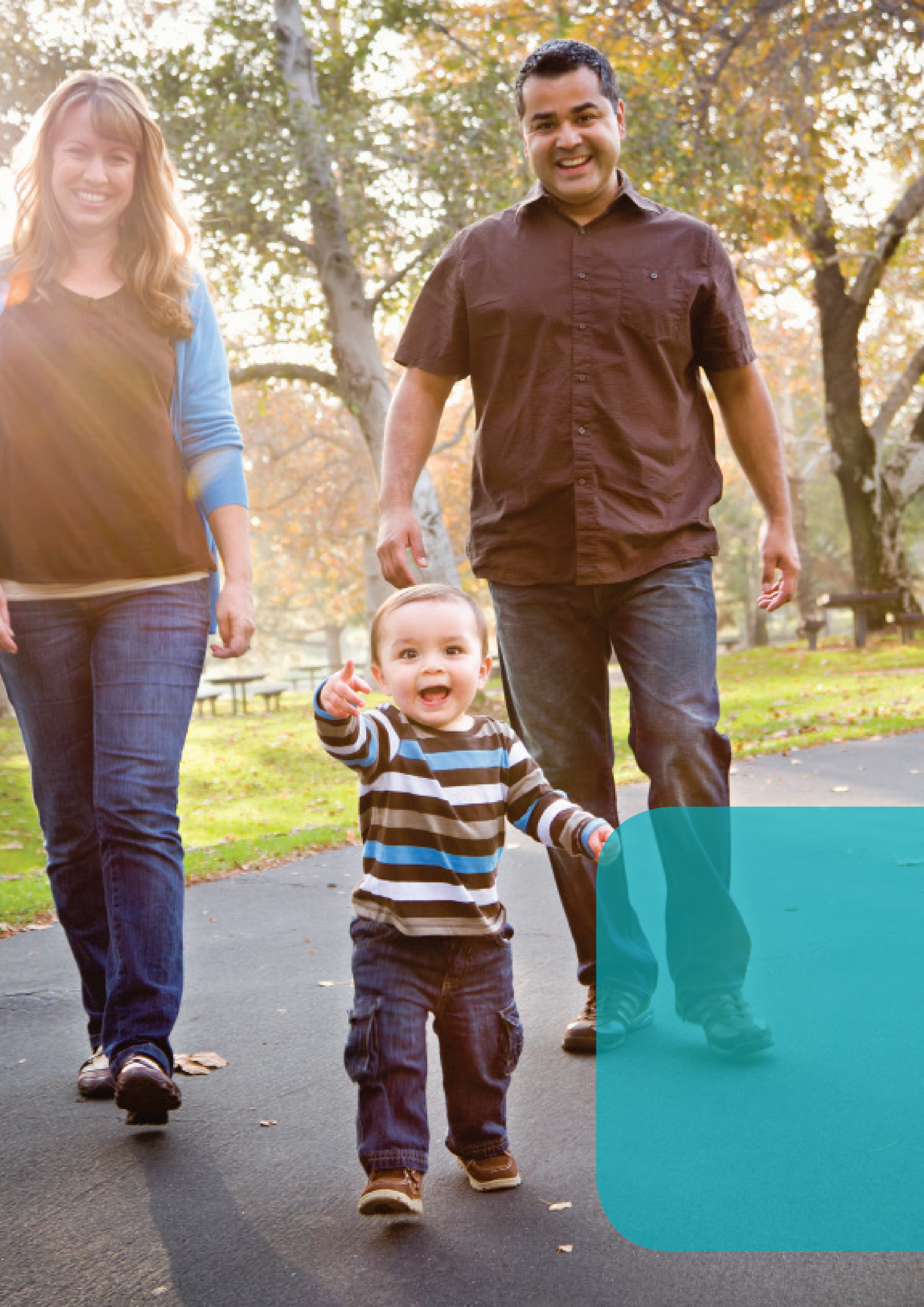

Supplement: Additional file 2: — Final guidelines on how partners can support one another to prevent perinatal depression and anxiety. (PDF 1173 kb) [file 12888_2016_721_MOESM2_ESM.pdf]
